# Variations in the expression of odorant binding and chemosensory proteins in the developmental stages of whitefly *Bemisia tabaci* Asia II-1

**DOI:** 10.1038/s41598-024-65785-9

**Published:** 2024-07-01

**Authors:** M. N. Rudra Gouda, S. Subramanian

**Affiliations:** Division of Entomology, Indian Agricultural Research Institute, New Delhi, 110012 India

**Keywords:** *Bemisia tabaci*, Asia II-1, Odorant binding protein, Chemosensory protein, Gene expression, Biotechnology, Bioinformatics

## Abstract

The cotton whitefly, *Bemisia tabaci*, is considered as a species complex with 46 cryptic species, with Asia II-1 being predominant in Asia. This study addresses a significant knowledge gap in the characterization of odorant-binding proteins (OBPs) and chemosensory proteins (CSPs) in Asia II-1. We explored the expression patterns of OBPs and CSPs throughout their developmental stages and compared the motif patterns of these proteins. Significant differences in expression patterns were observed for the 14 OBPs and 14 CSPs of *B. tabaci* Asia II-1, with OBP8 and CSP4 showing higher expression across the developmental stages. Phylogenetic analysis reveals that OBP8 and CSP4 form distinct clades, with OBP8 appearing to be an ancestral gene, giving rise to the evolution of other odorant-binding proteins in *B. tabaci*. The genomic distribution of OBPs and CSPs highlights gene clustering on the chromosomes, suggesting functional conservation and evolutionary events following the birth-and-death model. Molecular docking studies indicate strong binding affinities of OBP8 and CSP4 with various odour compounds like β-caryophyllene, α-pinene, β-pinene and limonene, reinforcing their roles in host recognition and reproductive functions. This study elaborates on our understanding of the putative roles of different OBPs and CSPs in *B. tabaci* Asia II-1, hitherto unexplored. The dynamics of the expression of OBPs and CSPs and their interactions with odour compounds offer scope for developing innovative methods for controlling this global invasive pest.

## Introduction

The olfactory system of insects, crucial for detecting odorants, oviposition, and mate recognition, is highly developed^1^. Our study focuses on characterizing insect olfactory-associated proteins, primarily odorant-binding proteins (OBPs) and chemosensory proteins (CSPs)^1^. These proteins play vital roles in insect olfaction, perceiving chemical signals and influencing behaviour^1^. Recognizing their significance, extensive research has been devoted to understanding them. Qiao et al.^2^ suggests their unique characteristics make them promising targets for novel pest control strategies.

Recognition of the chemical cues primarily involves the OBPs, which transport the odours to specific odorant receptors (ORs), resulting in the activation of the olfactory receptor neurons (ORNs), leading to behaviour manipulations^3^. The OBPs and CSPs located on the sensilla and other organs of insects break down the smell molecules, which is the first step in odour recognition^3^. The OBPs are water-soluble, acidic in nature, and have a globular shape. They convey semiochemical to olfactory receptors through antennal sensillum lymph, initiating interactions with cues like pheromones and host scents^4^. Their molecular weight ranges from 10 to 30 kDa, and they exhibit a distinct pattern of six conserved cysteine residues. Three interlocking disulfide bridges made of cysteine residues and other amino acids effectively bind and shield minute hydrophobic ligands, facilitating the formation of an odorant-binding pocket^5^. Pannure et al.^6^ highlight the diverse functions of OBPs in insects, including their involvement in pheromone signal transduction, host selection, and modulation of mating behaviour. They are also essential for the identification of cryptic species^7^.

The CSPs are multifunctional, small, soluble proteins found in great numbers in sensilla lymph^8^. CSPs, like OBPs, exhibit odour-binding properties. However, in contrast to OBPs, CSPs possess a higher degree of conserved nucleotide sequences and a lower degree of conserved cysteine residues, as reported by Pelosi et al.^9^. Jean-Francois^10^ suggests that CSPs in insects' non-sensory organs serve a broader array of functions when compared to OBPs. These functions include the distribution of pheromones, the solubilization of nutrition, and the development of resistance to pesticides. Bos et al.^11^ have noted that CSPs possess the capacity to act as effector proteins, thereby triggering the activation of physiological defence mechanisms in plants. In addition to the heads of insects, CSPs have been found in the thorax, abdomen, wings, and legs of numerous insects.

The expression of OBP and CSP genes varies with the developmental stages (egg, nymph, pupa, adult) of different insect species^12,13^. Understanding these variations is important because it can provide insights into how insects perceive and respond to their environment at different life stages. The differences in expressions of OBP/CSP genes in the developmental stages of the insects influence their behavioural physiology related to host recognition^14^, mating^15^, oviposition site recognition^16^, insecticide resistance^15^ etc. Information regarding the expression of these genes across different stages in *B. tabaci* has so far been restricted to the MEAM1 and MED genetic groups of the *B. tabaci* species complex^17,18^.

The present study aims to fill the knowledge gap with respect to OBPs and CSPs in *B. tabaci* Asia II-1, the predominant genetic group of whiteflies distributed throughout Asia. The present study focuses on the characterization of OBPs and CSPs of *B. tabaci* Asia II-1 by comparing their polypeptide sequence motifs and exploring the expression patterns of these two categories of olfactory proteins across the developmental stages of the insect, spanning from eggs to adults. The highly expressed OBPs and CSPs were further evaluated for their interaction with diverse odour compounds through in silico chemical competitive binding assays aimed at understanding their role in olfaction and host plant recognition. The comprehension of the molecular mechanisms underlying olfactory recognition has the potential to facilitate the identification of novel target sites for biorational molecules.

## Materials and methods

### Identification of putative OBPs and CSPs in *B. tabaci* Asia II-1

*B. tabaci* Asia II-1's whole genome sequence data was obtained by the National Institute for Biotechnology and Genetic Engineering's bioproject ID: PRJNA523911. The *B. tabaci* (Asia II-1) genome was then searched using the protein sequences of known OBPs and CSPs using the programme TBLASTN with an e-value cutoff of 10^−5^. The candidate OBP/CSP sequences were gathered from the sequences that met the requirements. By using BLASTX analysis with the non-redundant protein sequence (NR) at GenBank (http://www.ncbi.nlm.nih.gov/), putative OBP and CSP sequences were verified. We also cross-referenced the identified gene functions with the KEGG database^19^ (www.kegg.jp/kegg/kegg1.html) and subsequently submitted our findings to NCBI to gain accession. (Accession numbers for OBP and CSP sequences are given in Supplementary File 1; Table S1).

### Detection of OBPs and CSPs in developmental stages of *B. tabaci* AsiaII-1

The OBPs and CSPs were validated by performing PCR analysis followed by Sanger sequencing. For PCR reactions, RNA from the adult tissues of 200 adults in AsiaII-1 was extracted with Trizol reagent (Invitrogen, Carlsbad, CA, USA) according to the manufacturer’s instructions, and RNA purity and degradation were checked on 1% agarose gels and Nanodrop (Nabi™ Ultraviolet–Visible Nano Spectrophotometer from MicroDigital). Template cDNA was synthesised using the PrimeScript™ 1st Strand cDNA Synthesis Kit (TaKaRa, Dalian, China), and PCR amplification was carried out in a Bio-Rad C1000-thermal cycler (Bio-Rad Laboratories Inc, Berkeley, CA, USA). Primers were designed for each candidate OBP and CSP gene by using the Primer-BLAST tool at NCBI. A list of primers used is available in Supplementary File 1; Tables S2 and S3. PCR was performed under the following thermal profiles: 94 °C for 3 min; 35 cycles of 94 °C for 30 s; 52 °C for 30 s; and 72ºC for 1 min, followed by incubation at 72 °C for 10 min. The gels were run at 100 V for 1 h in TAE buffer (40 mM Tris–acetate, 1 mM ethylene-diamine-tetra-acetic acid (EDTA), pH 7.4). Gels were visualised under UV in the Gel Documentation System of the Alpha ImagerTM gel imaging system (Alpha Innotech, San Leandro, CA, USA). The products were sequenced by outsourcing the services of Barcode Bioscience in Bengaluru, India.

### Expression analysis OBPs and CSPs in developmental stages of *B. tabaci* Asia II-1

RT-qPCR analysis was carried out in CFX96 (BioRad® USA- Real-Time PCR Detection System) using gene-specific primers and SYBR Premix EX TaqTM (TaKaRa, Dalian, China) with three biological replicates. As a reference, two housekeeping genes from *B. tabaci*, β-actin (Accession number: EE600682) and EF-1 (AF071908), were used^20^, along with 28 pairs of primers for RTqPCR (Supplementary File 1; Tables S4 and S5). The 20 µl reactions used for the RT-qPCR contained 2 µl of cDNA (200 ng/ul), 10 µl of SYBR Premix Ex TaqTM (TaKaRa, Dalian, China), 1 µl each of forward and reverse primers (10 µM), 0.4 µl of Rox Reference Dye II, and 5.6 µl of nuclease-free water. The thermal cycling parameters were 95 °C for 30 s, 40 cycles of 95 °C for 5 s, and 62 °C for 34 s^18^. To confirm a single PCR product, all reactions were subjected to a melting curve analysis from 60 to 95 °C after the cycling process. The measurement of fluorescence during the 55–95 °C melting curve that followed was done to verify primer dimer peak absence and to identify a single gene-specific peak. For each primer examined, a distinct single peak was found. Negative controls involved substituting ddH_2_O for cDNA in non-template reactions. The Bio-Rad CFX Maestro analysis programme for the Bio-Rad CFX96 was used to examine the outcomes. The 2^−ΔΔCt^ technique was used to quantify the transcript levels of selected BtabOBPs and BtabCSPs^21^. Using Microsoft Excel-based software written in Visual Basic, the expression levels of these genes were compared to those of the two housekeeping genes^22,23^. Three biological replications were carried out for each sample, and the results of each biological replication were measured three times using different techniques. Using the SPSS Statistics 11.0 programme (SPSS Inc., Chicago, IL, USA), each target gene’s comparative analysis across different tissues was evaluated using a one-way test of variance (ANOVA), followed by Tukey’s honest significance difference (HSD) test. The values were shown as the mean standard error.

### Phylogenetic analyses

Two distinct phylogenetic analyses were conducted. The first analysis involved a dataset containing 14 sequences of OBP/CSP genes specific to *B. tabaci* Asia II-1 by neighbour-joining tree. The second analysis expanded the scope to include OBP/CSP genes from *B. tabaci* Asia II-1, MEAM1, and MED biotypes, along with the OBP/CSPs of other Hemipteran insects, to their evolutionary origin by the maximum likelihood approach. A comprehensive list of OBP/CSP gene sequences from Hemipteran species used in the phylogenetic analyses is furnished in Supplementary File 2. In both analyses, the ClustalW programme (http://www.clustal.org/omega/clustal-omega-1.2.2-win64.zip) was employed for sequence alignment, using default gap penalty values (gap opening: 10, extension: 0.2). Genetic distances were calculated using a p-distance model, and pairwise gap deletion was applied to handle gaps introduced during alignment. Subsequently, neighbour-joining phylogenetic trees were constructed using MEGA 11.0 (https://www.megasoftware.net/), drawing upon the approach by Tamura et al.^24^. To assess the robustness of the tree branches, bootstrap analysis was carried out by resampling aligned amino acid locations 1000 times. The resulting phylogenetic trees were presented in an unrooted format, allowing for a clear visualisation of the evolutionary relationships among the analysed sequences.

### Protein structure prediction of OBP8 and CSP4 of *B. tabaci* Asia II-1

SMART (simple modular architecture research tool; http://smart.emblheidelberg.de/) was used^25^ to predict the conserved domains of the OBPs and CSPs identified from *B. tabaci* Asia II-1. The conserved domain search service tool of NCBI was used to confirm the predictions by the SMART tool. Phyre2 (Protein Homology/Analogy Recognition Engine V 2.0) (http://www.sbg.bio.ic.ac.uk/~phyre2/html/page.cgi?id=index) was used to predict the structure of OBP8 and CSP4 proteins identified in *B. tabaci* Asia II-1. The intensive mode and ab initio techniques were used to perform complete modelling of the entire protein. Along with that, the possible functional role of the protein can also be known. We used a batch processing tool to process multiple sequences at once in expert mode. SWISS model (https://swissmodel.expasy.org/) and I-TASSER (https://zhanggroup.org/I-TASSER/) were used to generate a comparative model, but Phyre2 offered better models. The quality of our predicted models was checked using SAVES v5.0 (https://servicesn.mbi.ucla.edu/SAVES) tools, ProSA (https://prosa.services.came.sbg.ac.at/prosa.php/), and Qmean (https://swissmodel.expasy.org/qmean/). A diverse structural validation criterion was utilised to verify the precision and integrity of predicted protein structures. These criteria encompass identifying Ramachandran outliers (detecting unfavourable backbone conformations), recognising sidechain outliers (flagging problematic sidechain conformations), highlighting RSRZ outliers (indicating atypical stereochemical properties), quantifying steric clashes through clashscore assessment, and evaluating the quality of model fit to experimental data via Rfree measurements. Supplementary File 1; Fig. S1 provides details on the validation of the protein structures.

### Motif analysis

We conducted motif discovery and pattern analysis using a dataset consisting of 14 OBPs and CSPs from *B. tabaci* Asia II-1. To perform this analysis, we utilised MEME software, specifically version 5.5.1, through the online server accessible at http://meme-suite.org/index.html. For guiding the motif discovery process, we configured MEME with the following parameters: a minimum motif width of 6, a maximum motif width of 10, and a maximum limit of 6 motifs to be identified. Additionally, we adopted a zero-or-one occurrence per sequence (ZOOPS) distribution pattern during this analysis.

### In-Silico docking

The protein structure obtained above was downloaded in PDB format from the Protein Databank (PDB) structure, which was then prepared for docking using the AutoDock Vina plugin (https://vina.scripps.edu/wp-content/uploads/sites/55/2020/12/autodock_vina_1_1_2_win32.msi) for PyMOL^26^ (https://pymol.org/#download). The protein preparation was done by following the standard protocol^27^ by removing the co-crystallised ligand, selected water molecules, and cofactors. We have used some VOCs like β-ionone, p-cymene, β-ocimene, β-caryophyllene, neophytadiene, myrcene, β-pinene, α-pinene, cis-3-Hexen-1-ol, limonene, and azulene based on their perceived association with olfaction and host plant recognition of insects as documented in earlier literature (Supp Table S10). The energy-minimised ligand molecules were then used as input for AutoDock Vina in order to carry out the docking simulation. Next, the target protein file was prepared by leaving the associated residue with the protein by using the auto-preparation of the target protein file Auto Dock 4.2 (MGL Tools 1.5.6). The graphical user interface programme was used to set the grid box for docking simulations. The grid was set so that it surrounds the region of interest in the macromolecule. The docking algorithm provided with the software was used to search for the best docked conformation between ligand and protein. During the docking process, a maximum of nine conformers were considered for each ligand. The conformations with the most favourable (least) free binding energy were selected for analysing the interactions between the target receptor and ligands by Discovery Studio Visualizer (https://discover.3ds.com/discovery-studio-visualizer-download) and PyMOL. The ligands are represented in red, H-bonds, and the interacting residues are represented in ball and stick model representations (Figs. 6 and 7).

### Statistical analysis

ANOVA and Tukey’s test, were conducted using SPSS Statistics 16.0 (https://spss.software.informer.com/download/?cf206d24) to assess gene expression levels.

### Ethics approval statement

This study does not include any animals which require any kind of ethical clearance. We confirm that all experiments were performed in accordance with relevant guidelines and regulations of the Institute.

## Results

### Identification of OBPs and CSPs in *B. tabaci* Asia II-1

We have earlier identified the presence of 14 OBPs and 14 CSPs in the genome of *B. tabaci* Asia II-1. The details of the accession number of BtAsiaII-1 OBPs and CSPs, along with CDS information, are provided in supplementary file 1, table S1. We have identified three distinct domains namely, pfam PBP_GOBP, pfam PhBP, and pfam OS-D to be present in the OBPs. Notably, the pfam PBP_GOBP domain is predominantly observed within the OBPs. Conversely, we have noticed that the pfam OS-D domain is predominantly featured in the CSPs of *B. tabaci* Asia II-1. The details of domains present in the BtAsiaII-1 OBPs and CSPs are furnished in Supplementary File 1; Tables S6 and S7.

### Detection of BtAsiaII-1 OBP and CSP genes across the developmental stages

**Figure 1:**
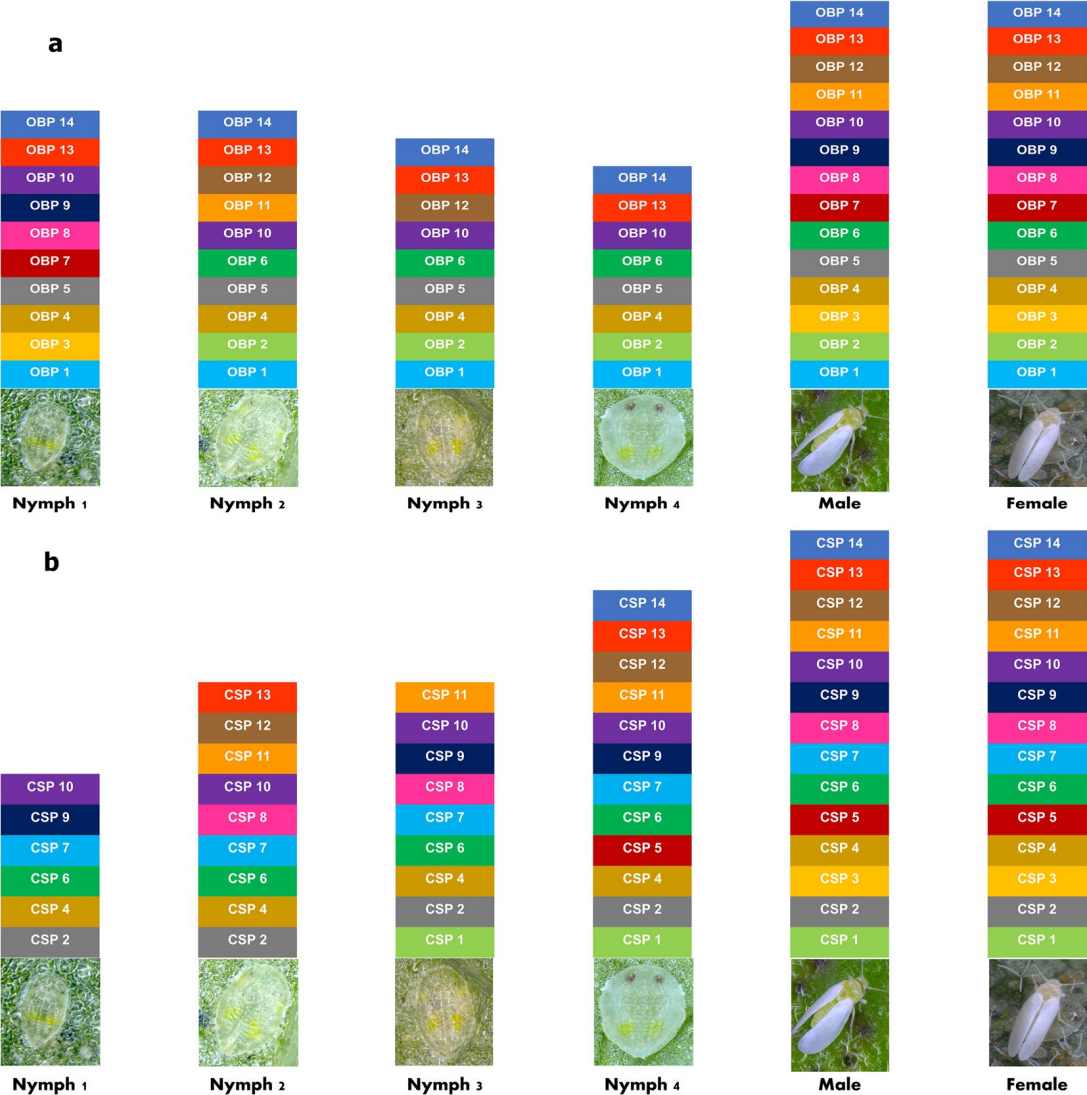
Representing OBP/CSP genes present across the different life stages of *B. tabaci* Asia II-1 cryptic species. Diagnostic PCR was performed for detection by gene specific primers. (**a**) OBP genes, (**b**) CSP genes.

We examined the following stages: egg, nymph 1, nymph 2, nymph 3, nymph 4 (prepupa), adult males, and adult females, to assess the presence of 14 OBPs and 14 CSPs through diagnostic PCR using gene-specific primers. Our analysis revealed that eggs do not contain any OBP/CSP genes. Specifically, OBP genes 1, 4, 5, 10, 13, and 14 were consistently present across all stages of *B. tabaci* Asia II-1. Among the nymphal stages, the 4th nymphal stage exhibited the fewest OBP genes, with eight, followed by the 3rd nymphal stage with nine genes. For further details, please refer to (Fig. 1a). In contrast, CSP genes 2, 4, 6, 7, and 10 were found to be present throughout all the growth stages of *B. tabaci* Asia II-1. The 1^st^ nymphal stage contained the lowest number of genes, with six, while the 2^nd^ and 3^rd^ nymphal stages had nine genes each. The 4^th^ nymphal instar stage had the highest number of CSP genes, with a total of 12. The presence of all the OBP/CSP genes were detected in the male and female adults of *B. tabaci* (Fig. 1b). It is to be noted that the polymerase chain reaction technique specifically identifies and amplifies genes with a certain copy number while disregarding those genes with lower copy numbers.

**Figure 2:**
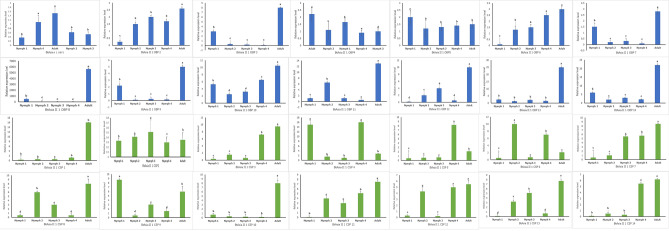
*B. tabaci* Asia II 1 OBPs and CSPs transcript levels in different stages as measured by RT-qPCR. A one-way analysis of variance (ANOVA) was employed, followed by Tukey’s honest significance difference (HSD) test for post-hoc analysis. Mean standard error values were utilized for representation. The expression levels were estimated using 2^−ΔΔCt^ method. The standard error for each sample is represented by the error bar, and the different letters (a, b, c, d, e) above each bar denote significant differences (p < 0.05).

**Figure 3:**
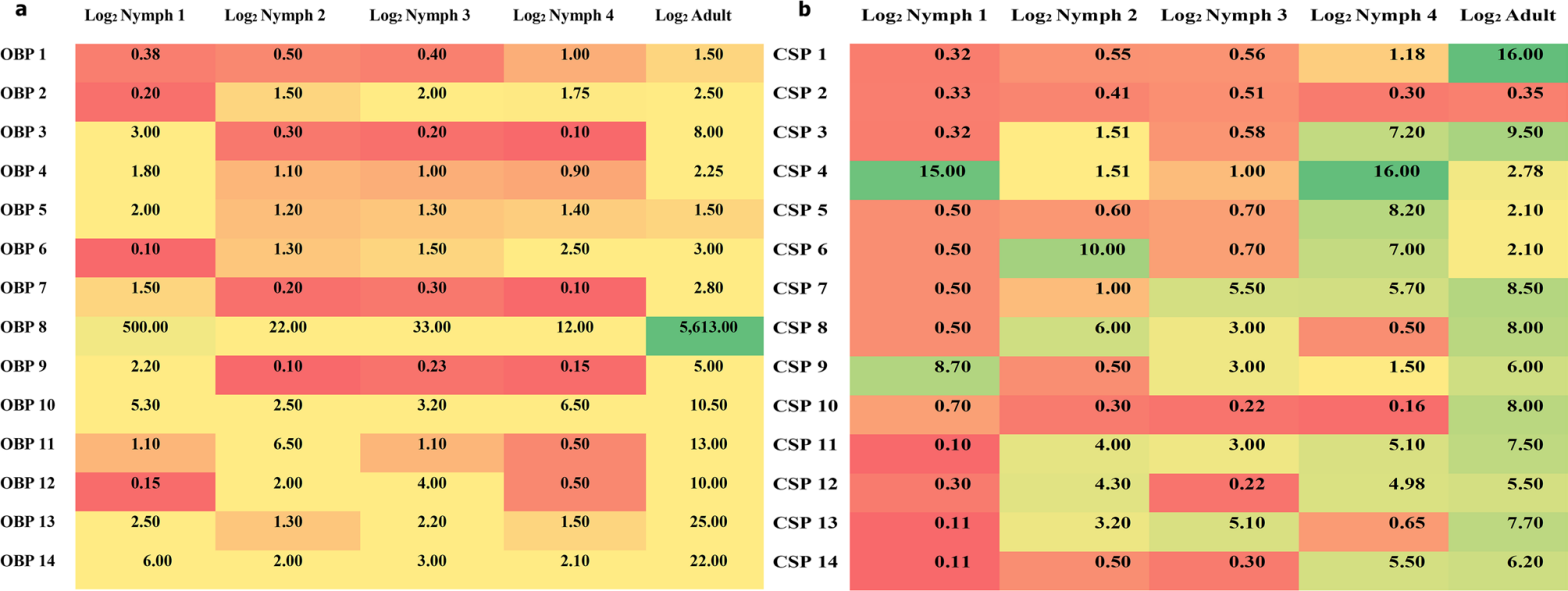
Heat map based on the expression profile of (**a**) OBP, (**b**) CSP genes across life stages of *B. tabaci* Asia II-1. The values indicated here are the respective fold changes of that gene in that particular stage. The expression of all the genes was significant at P < 0.05 with a Tukey test across different life stages. A colour scale was employed to denote significant expression levels, where red indicates low expression, yellow indicates moderate expression, and green signifies high expression.

The OBP/CSP genes not detected by PCR were further investigated for their expression levels using the quantitative PCR (qPCR) technique (Fig. 2). The expression of all these genes was statistically significant (P < 0.05) across different life stages. Notably, the OBP-8 consistently exhibited high expression levels in all developmental stages, with particularly elevated levels of expression in the adult stage. Next to OBP8, OBP10 showed relatively higher expression in nymphal stages one to four. The OBPs 14, 11, 12, and 10 were found to show higher levels of expressions in the nymphal stages first to fourth respectively. Whereas in the adults of *B*. *tabaci* Asia II-1, OBPs 13 and 14 were showing higher levels of expression. Conversely, the OBPs 6, 9, 3 and 7 were the least expressed in the nymphal instars first through fourth respectively. The CSPs 4, 6, and 7 exhibited relatively higher expressions in first, second and third instars respectively. While, CSP4 exhibited significantly higher expression during the fourth instar, CSP1 was showing the highest expression in the adults. Notably, CSP 4 had shown consistently higher expression than all other CSP genes across the nymphal stages and CSP 2 was showing the least expression across all life stages of *B. tabaci* Asia II-1. The heatmap representation (Fig. 3a and b) depicts the relative levels of expression of OBP and CSP genes in different life stages of *B. tabaci* Asia II-1. The relative fold change in expression of OBP8 and CSP4 in progressive growth stages with reference to stage one is presented in Fig S4. The OBP-8 showed 11-fold increase in expression in adults compared to nymphal stage one. Among the CSPs, the CSP4 showed progressive increase in gene expression through the nymphal stages.

### Motif structure and pattern analysis of BtAsiaII-1 OBPs and CSPs

**Figure 4:**
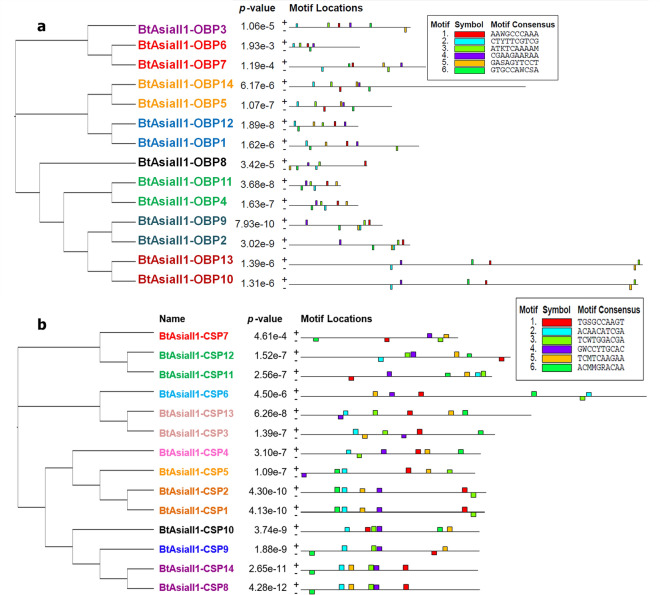
Motif pattern identified in OBP/CSPs of *B. tabaci* AsiaII-1. To guide motif discovery using MEME, we set the parameters as follows: minimum width of 6, maximum width of 10, and a limit of 6 motifs to be identified. Colour boxes in figures are motifs, and the pattern of that motif is indicated in the colour-coded box on the right. All the OBP/CSP motif patterns are less than p-value 0.0001 and are statistically significant. The length of the bar in each OBP/CSP indicates the length of the CDS region used in drawing motifs. The overall height of a stack indicates the degree of sequence conservation at that position. And on the left, the phylogenic relationship between OBP/CSP genes is shown. Cluster groups of OBP/CSPs are indicated on the right. (**a**) OBP, (**b**) CSP.

A comprehensive analysis of the motif structures and patterns of OBPs and CSPs of *B. tabaci* Asia II-1 was done. The amino acid codon sequences (CDS) of these proteins were used for motif analysis using the MEME tool. Importantly, each sequence demonstrated an E-value of less than 10, and the motif matches displayed position p-values lower than 0.0001. A total of nine motif patterns on the positive side and ten on the negative side were identified among the 14 OBPs of *B. tabaci* Asia II-1**.** The OBPs 3, 6, 8, and 7 exhibit unique motif patterns, while the remaining OBPs share similarities with other OBPs of *B. tabaci* Asia II-1 (Fig. 4a). As far as motif structures are concerned, six motifs were detected on both the positive and negative sides of BtAsiaII-1 OBPs (Fig. S2), and none of the OBPs displayed all six motifs. We identified 12 motif patterns on the positive side and 10 on the negative side of the CSPs of *B. tabaci* Asia II-1 (Fig. 4b). The majority of CSP genes had five motifs. The CSP 10 was found to display six motifs on the positive side, with no motifs detected on the negative side (Fig. S3). The CSPs 14, and 8, as well as CSPs 1 and 2, shared similar motif patterns between them. The remaining 10 CSPs exhibit unique patterns. Comparatively, CSPs have a greater number of unique patterns than OBPs (Fig. 4b).

### Phylogenetic investigation of *B. tabaci* Asia II-1 OBPs and CSPs

**Figure 5:**
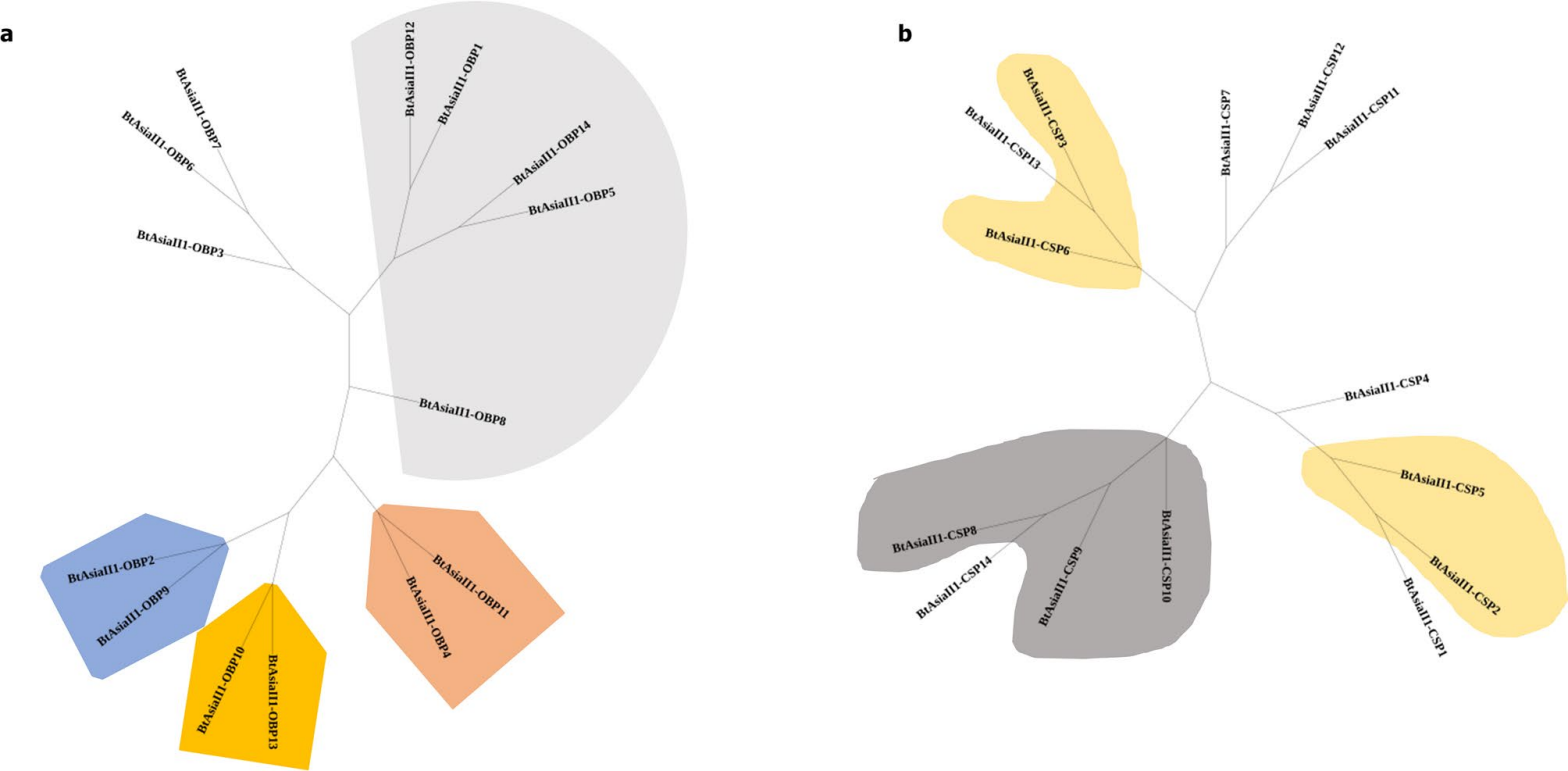
(**a**) Phylogenetic study of *Bemisia tabaci* Asia II 1’s odorant-binding proteins (OBPs). Based on the nucleotide sequences of *Bemisia tabaci* Asia II-1, a neighbor-joining tree was created for the odorant-binding proteins (OBPs). The 1000 replications used to obtain the bootstrap values are indicated on the nodes. Genes present on chromosome 3 are indicated in grey, blue on chromosome 2, gold on chromosome 6, and an orange accent on chromosome 10. (**a**) Phylogenetic study of *Bemisia tabaci* Asia II 1’s chemosensory proteins (CSPs). Based on the nucleotide sequences of *Bemisia tabaci* Asia II-1, a neighbour-joining tree was created for the chemosensory proteins (CSPs). The 1000 replications used to obtain the bootstrap values are indicated on the nodes. Genes present on chromosome 7 are indicated in grey or light cream on chromosome 6.

We successfully mapped the positions of all OBP genes in the *B. tabaci* genome except for BtAsiaII1-OBP3. Our findings reveal that the 13 OBP genes tend to cluster on four chromosomes. Majorly six OBP genes were clustered on chromosome 3. CSPs were primarily clustered on two chromosomes, with six CSP genes on chromosome 6 and four on chromosome 7 (details in Tables S8 and S9 of Supplementary File 1). We constructed the neighbour-joining trees to provide a visual representation of the relationships between the OBPs and CSPs of *B. tabaci* Asia II-1 (Fig. 5a and b). The unrooted tree of OBPs is found to have a major clade consisting of four genes, namely OBP 1, 5, 12, and 14, and interestingly, all of these four genes are clustered on chromosome number three. The OBP 8 is presented as a uniclade among the OBPs of Asia II-1. Eight of 14 CSP genes branched into four clades, each containing two CSP genes, while six CSPs did not share any similarity with other clades. CSP 4 and CSP 7, located separately on chromosomes nine and four, did not share a clade with other CSPs in the phylogenetic tree. A phylogenetic tree was constructed by including the sequences of OBPs/CSPs identified in this study along with that of OBP and CSP sequences of other Hemipteran insects and *B. tabaci* genetic groups MED and MEAM1. This analysis delineates the evolutionary trajectory of hemipteran OBPs and CSPs (Figs. S5 and S6). Additionally, we traced the evolutionary progression of OBPs and CSPs within different genetic groups of *B. tabaci* using a neighbourhood joining tree (Figs. S7 and S8).

### Molecular docking studies

The interaction between OBP8/CSP4 and a certain volatile organic compound (VOC) was investigated by in silico docking analysis. The binding energy was used as a criterion to evaluate the affinity between these two genes and the selected VOCs. We have chosen 10 VOCs that have earlier been documented as attractants or repellents against *B. tabaci,* as per the available literature (Supp Table S10). Our objective was to optimise these interactions to achieve the most favourable binding with the lowest energy requirements. Our results reveal that all the ligand compounds established robust bonds with one or more amino acids within the active pockets of the OBP8/CSP4. Furthermore, these compounds exhibited binding energies within a range of − 3.8 to − 5.5 kcal/mol (Table 1).

**Table 1:** Binding energy values (Kcal/mol), obtained by docking simulations, between putative plant OBP8 and CSP4 and VOCs.

Cas id	Compound name	Binding energy (kcal/mol) OBP8	Cas id	Compound name	Binding energy (kcal/mol) CSP4
*79-77-6*	β-Ionone	− 5.0	*79*-*77-6*	β-Ionone	− 5.1
*99-87-6*	p-cymene	− 5.0	*99*-*87-6*	p-Cymene	− 4.6
13877-91-3	β-Ocimene	− 4.8	13877-91-3	β-Ocimene	− 4.3
87-44-5	β-caryophyllene	− 5.3	275-51-4	Azulene	− 4.9
13131714	2,4-Phytadiene	− 5.2	87-44-5	β-caryophyllene	− 5.3
123-35-3	Myrcene	− 4.8	13131714	2,4-Phytadiene	− 4.8
19902-08-0	β-Pinene	− 5.5	123-35-3	Myrcene	− 4.8
80-56-8	α-pinene	− 5.2	80-56-8	α-pinene	− 5.2
928-96-1	Cis-3-Hexen-1-ol	− 3.8	928-96-1	Cis-3-Hexen-1-ol	− 3.8
138-86-3	Limonene	− 5.2	138-86-3	Limonene	− 5.1

**Figure 6:**
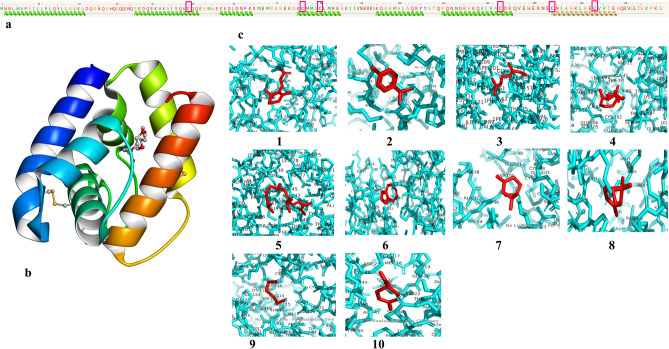
(**a**) Amino acid sequence of the CDS region of OBP8 green helix indicates alpha chains. (**b**) Secondary structure of the OBP8 CDS region c: ligand interaction with the binding pocket in protein (1, β-Ionone; 2, p-cymene; 3, β-Ocimene; 4, β-caryophyllene; 5, 2,4-Phytadiene; 6, Myrcene; 7, (+)-β-Pinene; 8, α-pinene; 9, cis-3-Hexen-1-ol and 10, Limonene).

**Figure 7:**
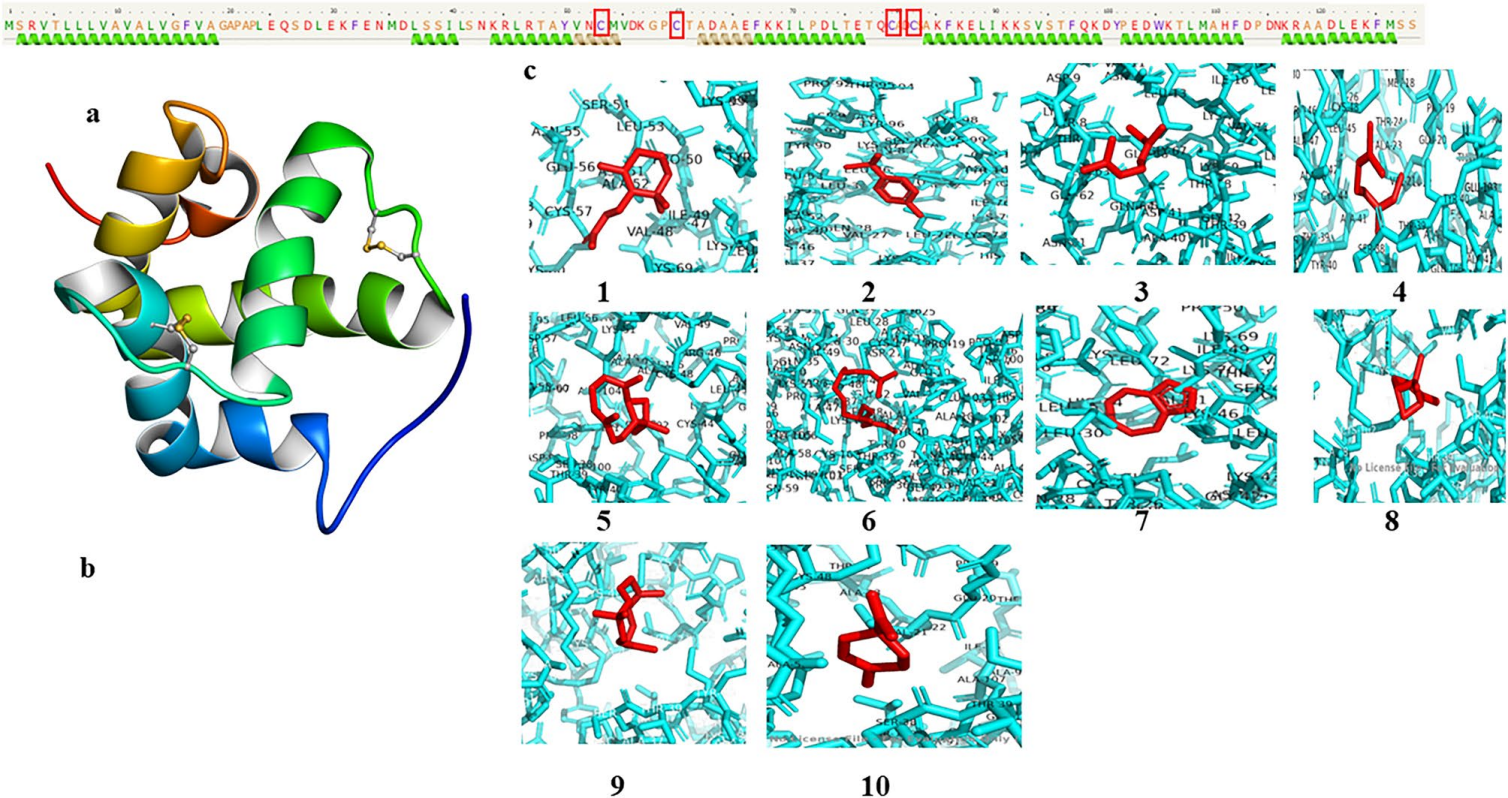
(**a**) Amino acid sequence of CDS region of CSP4 green helix indicates alpha chains, (**b**) secondary structure of CSP4 CDS region c: ligand interaction with the binding pocket in protein (1, β-Ionone; 2, p-cymene; 3, β-Ocimene; 4, Azulene; 5, β-caryophyllene; 6, 2,4-Phytadiene; 7, Myrcene; 8, α-pinene; 9, cis-3-Hexen-1-ol; and 10, Limonene).

β-caryophyllene demonstrated a high binding affinity to both CSP4 and OBP8, with the least binding energy of − 5.3 kcal/mol. In contrast, cis-3-hexen-1-ol displayed a lower binding affinity to both OBP8 and CSP4 proteins, as revealed by a lower binding energy value of − 3.8 kcal/mol (Figs. 6 and 7). Apart from these, β-pinene exhibited a high binding score of − 5.5 kcal/mol to OBP 8. Among the compounds screened, VOCs like p-cymene, 2,4-Phytadiene, α-pinene, and limonene were found to have binding energies greater than − 5 kcal/mol to OBP8. β-Ionone, α-pinene, and limonene were showing relatively higher binding affinity to CSP 4, as revealed by their binding energy values > − 5.0 kcal/mol.

## Discussion

The globally distributed cotton whitefly, *B. tabaci*, encompasses 46 cryptic species, with 16 found in Asia^28^. Despite Asia II-1’s prevalence, knowledge gaps persist regarding its OBPs and CSPs. Our study examined these proteins’ polypeptide sequence motifs and their expression across developmental stages. We assessed highly expressed OBPs and CSPs for interactions with odour compounds via in silico docking, shedding light on their roles in olfaction and host recognition. OBPs and CSPs play crucial roles in insect olfaction by transporting ligands to activate chemosensory transmembrane proteins^1,9,29^. They influence insect behaviour and physiological adaptations, including food-seeking, reproduction, and pesticide resistance^1,15,29,30^. Prior research on OBP diversity in *B. tabaci* was confined to the MEAM1 and MED genetic groups. Our study provides insight into stage-specific expression of OBPs and CSPs from *B. tabaci* Asia II-1 prevalent widely in Asia. Domain analysis revealed that most of the OBPs, excluding OBP10 and OBP13 possess PBP_GOBP superfamily domain suggesting their involvement in odorant binding as per gene ontology. All the CSP proteins have OS-D superfamily domain implying their likely involvement in chemosensory signal transduction^31^.

Phylogenetic analyses offer valuable insights into the evolutionary relationships between the OBPs and CSPs of different Hemipteran insects. Our analysis revealed that the OBP8 of *B. tabaci* Asia II-1 grouped with the OBP8 of MED and MEAM-1 and remained separated from the OBPs of other Hemipteran insects. It shows that OBP8 may be an ancestral gene in the evolution of all other odorant-binding proteins of *B. tabaci*. Among the newly reported OBPs (OBP 9 to 14) from *B. tabaci* AsiaII-1, the OBPs 10 and 13 formed distinct clusters without sharing clades with any other Hemipteran OBPs, implying its exclusivity to the Asia II-1 genetic group of *B. tabaci*. Our analysis also revealed that the OBP 8 and CSP4, located respectively on chromosomes three and nine of *B. tabaci,* branched as distinct clades, indicating their uniqueness. The neighbourhood joining analysis of the OBPs of *B. tabaci* cryptic species also shows that OBP8 remains as a uniclade in the tree without having any shared branches with other OBPs of *B. tabaci* Asia II-1.

The CSPs of *B. tabaci* cryptic species grouped together and formed separate clades from those of other hemipteran CSPs. However, the CSP4 of Asia II-1/MED, along with the CSP7 of MEAM1, constituted a unique clade. Branching as a distinct clade and unique motif pattern of CSP4 coupled with the significantly higher expression levels in developmental stages in *B. tabaci* Asia II-1, MED^18^, and MEAM1^17^ strongly suggest that CSP4 may play a vital role in the behavioural physiology of *B. tabaci.*

Chromosomal location and protein motif analysis are valuable tools for investigating gene evolution, including events like duplication, reversal, or skipping, and for assessing functional conservation^32,33^. In general, approximately half of the Asia II-1 OBPs and CSPs cluster on chromosomes three, six, and seven, often sharing the same loci on the chromosome. This clustering was likely to have resulted from the high conservation of these gene families in *B. tabaci* Asia II-1. Notably, the motif patterns display partial conservation among representatives within the same cluster. The evolution of the OBP gene family seems to follow the birth-and-death model, involving pseudogenization or functional divergence of duplicate genes during duplication events^34,35^. However, the adjacent genes on the same chromosome may be involved in analogous functions, as observed in other organisms like the fire ant *Solenopsis invicta,* wherein certain genes on the social chromosome play a critical role in behavioural modulation between monogyne and polygyne colonies^36^.

Unlike the OBPs, which form multiple clusters with fewer genes within each cluster, the CSPs *B. tabaci* Asia II-1 primarily formed two clusters, indicating a potential origin through gene duplication. The CSP2 and CSP3 clustered together on chromosome number six (Table S9) of *B. tabaci* were associated with insect defence^15^. However, the OBPs 1 and 8 clustered together on chromosome number three were found to be associated, respectively, with different functions like identification of the oviposition site^16^ and host preference^37^. This supports the birth-and-death model of the evolutionary origin of OBPs. However, further studies may elucidate the functional roles of closely and distantly located OBPs and CSPs on the chromosomes of *B. tabaci*.

Understanding the expression patterns of OBPs and CSPs at different life stages is crucial for uncovering their roles in various adaptations such as feeding preferences, mate-seeking behaviour, and pesticide resistance^13,38^. Interestingly, the BtAsiaII-1 OBPs/CSPs were not detected in the egg stage, suggesting that they may not play a role in embryonic development, unlike in some other insects like *Galeruca daurica,* where a specific OBP, *i.e.,* GdauOBP28, was highly expressed during the egg stage^39^. Earlier studies have shown that OBPs like 3 and 8 contribute to the orientation of insects towards their host plants at medium or short distances in the *B. tabaci* MED and MEAM1 genetic groups^37,40,41^. The OBPs like 3,4,5,7,8,9,10,13, and 14 were observed to show good expression in the first instar nymphs, the stage wherein the insect starts feeding by probing their stylets into the phloem tissues of the host plant. We speculate that these OBPs may be primarily involved in host finding and feeding in *B. tabaci*. It may be probable that whitefly *B. tabaci* may recruit different OBPs for recognition of chemical cues from host plants, as this pest is recorded as a polyphagous pest with a host range of more than 200 plants. Detailed studies are needed to ascertain the functional roles of these OBP genes. An increased number of OBPs have been identified in non-sensory tissues such as the pheromone glands, wings, legs, fat body, and salivary glands^29^. They have roles in pheromone delivery, host adaptation, development, reproduction, and insecticide resistance^38^. OBP 27 in *Spodoptera frugiperda*^13,38^ and OBP 56a in *Phormia regina*^30^ have roles in binding and transporting fatty acids. OBP 27 is highly expressed in the reproductive organs of males and is involved in the mating of males in *S*. *frugiperda*^38^. AeOBP22 might transport or sequester a pheromonal component during mating^42^. OBP10 in *Helicoverpa armigera* and *Helicoverpa assulta* might act as carriers of oviposition deterrents and mediate the dissemination of eggs in species with larval cannibalism^43^. Further studies are needed to unravel the non-sensory role of OBPs in the nymphal stages of whitefly *B. tabaci*.

It is significant to note that OBP 8 displayed significant levels of gene expression in all the developmental stages and particularly a higher expression (5613 log_2_ fold change) during the adult stage of *B. tabaci* Asia II-1 (Fig. 5a). This extremely high level of expression underscores the functional significance of OBP8 in the lifecycle of *B. tabaci* Asia II-1. Higher levels of expression of OBP8 were earlier recorded in adults of the *B. tabaci* genetic groups MED^37^ and MEAM1^17^. Consistently higher expression of OBP8 in different genetic groups suggests that the OBP8 may play a crucial role in survival, host recognition, and feeding, and it may as well be involved in other physiological functions of *B. tabaci.* The role of OBP8 in host recognition in adults of *B. tabaci* MED had earlier been documented by Wang et al.^37^. Barring OBP5, all other OBPs in the present study have shown significantly higher expression in adults, and eventually they may be associated with host recognition, feeding, and reproductive physiology in *B. tabaci* Asia II-1. Contrary to our results, He et al.^14^ reported that OBP5showing higher levels of expression in the adult *B. tabaci* MED was found to be associated with host recognition. Such variations in expression levels of OBPs may be attributed to biotype-specific variations of OBP genes in *B. tabaci* cryptic species. An earlier study reported that biotype-specific signatures in CSPs 1, 2, and 3 in the MED and MEAM1 genetic groups of *B. tabaci,* was leading to functional variations^15^. The OBPs 1 and 4 expressed higher in adults of *B. tabaci* were implicated in oviposition site detection^16^. The OBPs 8 and 3 of *B. tabaci* were found to be associated with host findings by Wang et al.^37^ and Shi et al.^41^. Most of the BtAsiaII-1 OBPs showed low levels of expression during the fourth instar/prepupal stage, which is incidentally a non-feeding stage of the insect^44^.

Results of our study show that all the BtAsiaII-1 CSPs except CSP 9 exhibited significantly higher expression in the prepupal and adult stages, suggesting their role in the reproductive physiology of *B. tabaci*, as reproductive organ development occurs during the prepupal stage^44^, and these may be involved as well in mating and oviposition during adulthood. The lower expression of CSPs in the first instar nymph indicates that they may not be primarily involved in feeding. Expression of CSPs barring CSP 4 and 9 was relatively higher in the second and third nymphal stages compared to the first nymphal stage and adulthood (Figs. 4 and 5) implying that these CSPs may be associated with other physiological functions. Variation in expression of CSPs has earlier been reported in the MEAM1 and MED genetic groups of *B. tabaci*^15,16,45^. The functional role of some of the CSPs including 1, 2, 3, and 11, have earlier been deduced in these *B. tabaci* genetic groups. CSP1 was found to be associated with insecticide resistance^15^, while CSP2 was implicated in olfaction host plant recognition and oviposition preference^15,46^. The CSP3 expressed at high levels in nymphs of B and Q biotypes of *B. tabaci* was found to play a role in odour recognition from host plants^15^. The CSP-11 was associated with reproduction^45^. However, there has been no report on the functional characterization of OBPs and CSPs in *B. tabaci* Asia II-1, the widely distributed in Asia.

Significantly high expression of genes like OBP8 and CSP4 in the developmental stages of *B. tabaci* Asia II-1 prompted us to explore its possible role in olfaction. We deduced the predicted protein models for OBP8 and CSP4 and the predicted structures conform to the Ramachandran Plot. The secondary structure predictions for OBP8 and CSP4 yielded confidence values of 99.9% and 100%, respectively. It is significant to note that it showed 200-to-5000-fold increased expression compared to other OBPs in the adult stage, implying its significance in eliciting behavioural responses in the whitefly, *B. tabaci*. Probably, it may be involved in host recognition or play a role in the growth and developmental physiology of the insect. Detailed studies are needed to ascertain its functional role in *B. tabaci.*

We conducted molecular docking with 10 ligands for both OBP8 and CSP4 in *B.* tabaci Asia II-1. These ligands included green plant volatiles or HIPVs known to interact with insects in olfaction and host plant recognition. OBP8 showed strong binding with limonene, α-pinene, β-pinene, p-cymene, β-ionone, β-caryophyllene, and 2,4-Phytadiene (> − 5 kcal/mol), while β-ocimene and myrcene exhibited moderate binding, and cis-3-Hexen-1-ol had low binding affinity. Previous studies implicated OBP1 and OBP4 in oviposition site selection due to their strong binding to β-ionone, suggesting its role as a cue for host selection by *B. tabaci.*

Our findings indicated a significant binding affinity of β-ionone to both OBP8 and CSP4, with binding energies of − 5 kcal/mol and − 5.1 kcal/mol, respectively. Considering the abundant expression of OBP8 in the adult stage of *B. tabaci* and its robust affinity for β-ionone, we may speculate its possible role in host recognition or reproductive functions. α-pinene and limonene also exhibited high binding energy values exceeding − 5 kcal/mol. Our results are in concordance with the findings of He et al.^14^ who reported that these compounds enhance whiteflies’ ability to locate host plants. Furthermore, α-pinene had been implicated in eliciting a positive response from virus-infested plants to *B. tabaci* facilitating the rapid transmission of cucumber mosaic virus^47^, tomato leaf curl virus^48^ and cotton leaf curl virus^49^. Myrcene bound strongly to both OBP8 and CSP4 (− 4.8 kcal/mol), while β-ocimene showed moderate affinities to OBP8 (− 4.8 kcal/mol) and CSP4 (− 4.3 kcal/mol). Liu et al.^50^ demonstrated the attractancy of these compounds to *Encarsia formosa* and whiteflies. Other VOCs like β-caryophyllene, p-cymene, and 2,4-phytadiene also showed strong binding affinity to OBP8 (> − 5 kcal/mol), while β-caryophyllene and azulene showed good binding to CSP4 (− 5.3 kcal/mol and − 4.9 kcal/mol, respectively).

As of now, only a few studies have been taken on the functional characterization of OBPs and CSPs in the whitefly, *B. tabaci*. Our study provides an insight into expression patterns of these OBPs/CSPs across all the developmental stages of *B. tabaci* Asia II-1, hitherto unexplored. The differential expression patterns of certain BtAsiaII-1 OBPs and CSPs call for unravelling their functional role. Significantly high expression of OBP8 and CSP4 consistently across the developmental stages of *B. tabaci* Asia II-1 prompted us to explore its possible role in olfaction through *in-silico* docking analysis with volatile organic compounds having perceived roles in olfaction and host plant recognition. The *in-silico* analysis particularly identified that compounds such asα-pinene, β-pinene, 2,4-Phytadiene, β-caryophyllene, and β-ionone modulated the orientation behaviour of *B. tabaci* Asia II-1. However, detailed in vitro ligand binding studies with different OBPs and CSPs may confirm the assertions made in this study. Moreover, functional validation can be done through use of tools such as RNAi or CRSPR-Cas9 evaluation systems. Despite these limitations, our study lays the groundwork for future research into the functional roles of OBPs and CSPs in *B. tabaci* Asia II-1. Unravelling the functional role of OBP/CSPs may lead to development of novel control strategies for this global invasive pest.

### Supplementary Information

Supplementary Information 1.

Supplementary Information 2.

## Data Availability

All data generated or analysed during this study are included in this article (and its Supplementary Information files). The details of OBP and CSP gene sequences of *B. tabaci* AsiaII-1 generated in this study have been deposited with NCBI Nucleotide Database vide accession numbers: OQ304007 to OQ304034.
