# Supplementary material for: Variations in the expression of odorant binding and chemosensory proteins in the developmental stages of whitefly Bemisia tabaci Asia II-1
Source: Sci Rep. 2024 Jul 1;14:15046. doi: 10.1038/s41598-024-65785-9 (PMC11217293; doi:10.1038/s41598-024-65785-9)

**Variations in the Expression of Odorant binding and Chemosensory Proteins in the Developmental Stages of Whitefly *Bemisia tabac*i Asia II-1**

**M. N. Rudra Gouda^1^ and S. Subramanian^2#^**

**# Corresponding author:** [**entosubra@yahoo.co.in**](mailto:entosubra@yahoo.co.in)

**M. N. Rudra Gouda^1^**

Research Scholar, Division of Entomology/, Indian Agricultural Research Institute, New Delhi- 110012, India

e-mail: [rudragoudamn@gmail.com](file:///C:\Users\DELL\Desktop\rudragoudamn@gmail.com)

Orcid Id: 0000-0002-1644-4226

**Sabtharishi Subramanian^#2^**

Principle Scientist, Division of Entomology, Indian Agricultural Research Institute, New Delhi- 110012, India

e-mail: [entosubra@yahoo.co.in](mailto:entosubra@yahoo.co.in)

Phone: 8076595366

Orcid Id: 0000-0001-8337-9666

**Table. S1: List of accession number of *Bemisia tabaci* Asia II-1 OBPs and CSPs along with CDS information**

| **Gene** | **Accession** | **Sequence length in bp** | **CDS**  **nt/aa** | **Start** | **End** |
| --- | --- | --- | --- | --- | --- |
| 1 | 2 | 3 | 4 | 5 | 6 |
| **OBP genes** | | | | | |
| BtAsiaII1-OBP1 | OQ304010 | 800/complete | 426/142 | 78 | 503 |
| BtAsiaII1-OBP2 | OQ304011 | 744/partial | 744/248 | 1 | 744 |
| BtAsiaII1-OBP3 | OQ304012 | 747/partial | 747/249 | 1 | 747 |
| BtAsiaII1-OBP4 | OQ304013 | 426/partial | 426/142 | 1 | 426 |
| BtAsiaII1-OBP5 | OQ304014 | 633/partial | 633/210 | 1 | 633 |
| BtAsiaII1-OBP6 | OQ304015 | 435/partial | 435/145 | 1 | 435 |
| BtAsiaII1-OBP7 | OQ304016 | 864/partial | 864/287 | 1 | 864 |
| BtAsiaII1-OBP8 | OQ304017 | 480/partial | 480/160 | 1 | 480 |
| BtAsiaII1-OBP9 | OQ304007 | 575/partial | 536/178 | 1 | 536 |
| BtAsiaII1-OBP10 | OQ304018 | 2151/complete | 740/246 | 171 | 911 |
| BtAsiaII1-OBP11 | OQ304008 | 318/partial | 318/106 | 1 | 318 |
| BtAsiaII1-OBP12 | OQ304009 | 426/partial | 426/142 | 1 | 426 |
| BtAsiaII1-OBP13 | OQ304019 | 2177/complete | 740/246 | 172 | 912 |
| BtAsiaII1-OBP14 | OQ304020 | 1456/complete | 636/211 | 99 | 734 |
| **CSP genes** | | | | | |
| BtAsiaII1-CSP1 | OQ304023 | 393/partial | 393/131 | 1 | 393 |
| BtAsiaII1-CSP2 | OQ304024 | 396/partial | 396/132 | 1 | 396 |
| BtAsiaII1-CSP3 | OQ304025 | 414/partial | 414/138 | 1 | 414 |
| BtAsiaII1-CSP4 | OQ304026 | 384/partial | 384/128 | 1 | 384 |
| BtAsiaII1-CSP5 | OQ304027 | 372/partial | 372/124 | 1 | 372 |
| BtAsiaII1-CSP6 | OQ304028 | 738/partial | 738/246 | 1 | 738 |
| BtAsiaII1-CSP7 | OQ304029 | 336/partial | 336/112 | 1 | 336 |
| BtAsiaII1-CSP8 | OQ304021 | 382/partial | 382/124 | 1 | 382 |
| BtAsiaII1-CSP9 | OQ304030 | 381/partial | 381/123 | 1 | 381 |
| BtAsiaII1-CSP10 | OQ304031 | 381/partial | 381/123 | 1 | 381 |
| BtAsiaII1-CSP11 | OQ304032 | 408/partial | 408/136 | 1 | 408 |
| BtAsiaII1-CSP12 | OQ304033 | 453/partial | 453/151 | 1 | 453 |
| BtAsiaII1-CSP13 | OQ304034 | 492/partial | 492/164 | 1 | 492 |
| BtAsiaII1-CSP14 | OQ304022 | 378/partial | 378/126 | 1 | 378 |

**Table. S2: Primers used for PCR analysis of BtAsiaII1 OBP genes**

| Gene | Primer (5′ to 3′) |
| --- | --- |
| BtAsiaII1-OBP1 | F: TACCCATGCTGAAATGGACC |
|  | R: GTTGCGTGAACGATTTTCTG |
| BtAsiaII1-OBP2 | F: GTGTGTCTACAACGAAACGG |
|  | R: CACTGGCATATTTGGGCATT |
| BtAsiaII1-OBP3 | F: TGGACATCTTTTCCTGCGAG |
|  | R: CTTTCCACTCGCTATCAGGG |
| BtAsiaII1-OBP4 | F: GTTCTTTTCGTTTTCGCCTG |
|  | R: AAGGAACACTTCATACCCGC |
| BtAsiaII1-OBP5 | F: AAAATTGGAGAGGGTGGTCG |
|  | R: CCCCTTCAGAGTAAAGTCGG |
| BtAsiaII1-OBP6 | F: AGAGGTGTGTTTCGCACTC |
|  | R: GGTAGCATCTAAAATGGCGTC |
| BtAsiaII1-OBP7 | F: ACGATAGATACTGCTCCTCC |
|  | R: GTCGAGCATCTTCTGTTAGC |
| BtAsiaII1-OBP8 | F: ACCAAGGACATCAAGGACAG |
|  | R: GTTAAGCCAACCCTTTGAGC |
| BtAsiaII1-OBP9 | F: GACAACCCAAGCAAATGGTG |
|  | R: GACATTCCTCACTGTTGGTC |
| BtAsiaII1-OBP10 | F: GAAGACGGTCAAGAAAACCG |
|  | R: CATTCCAAACCCTCATTTCAAC |
| BtAsiaII1-OBP11 | F: CGAAGCAAAAGGTAGCGG |
|  | R: GCCAAGGAACACTTCATACC |
| BtAsiaII1-OBP12 | F: CTTTTCGTCGTCACCTTGTC |
|  | R: GACACGAGCCAAGATTTTCC |
| BtAsiaII1-OBP13 | F: GTGATCTCGCTCGTTTATCG |
|  | R: TACCGTACTTTCGCCTGTAG |
| BtAsiaII1-OBP14 | F: AAGTTTCGCCAACTGCAAAG |
|  | R: TGAGGGAAACCTGTGTCATC |

**Table. S3 Primers used for PCR analysis of BtAsiaII1 CSP genes**

| Gene | Primer (5′ to 3′) |
| --- | --- |
| BtAsiaII1-CSP1 | F: CCTGCCGAAGACAAATACAC |
|  | R: GAGGTACTCTTGAACGAGGG |
| BtAsiaII1-CSP2 | F: TGCCGAAGACAAATACACG |
|  | R: CCTTGAGGTACTCTTGAACG |
| BtAsiaII1-CSP3 | F: CACGAGGAAAGTACGAAGG |
|  | R: CCGCACTTGTTGGATAGAGC |
| BtAsiaII1-CSP4 | F: GACAAAGGACCCTGTACTGC |
|  | R: CTAAATCGGCTGCTCTCTTG |
| BtAsiaII1-CSP5 | F: GTTTGTGTTGTGCGTAGTGG |
|  | R: GAACTTGGCGTACTGCTCTC |
| BtAsiaII1-CSP6 | F: GGTGCCTGACTTACTTCCAA |
|  | R: GGTTGTTCGTGTTGTTGACC |
| BtAsiaII1-CSP7 | F: TGAGCTTCTTGTGCCTGTC |
|  | R: GGACCTTCTGGATCTGTCG |
| BtAsiaII1-CSP8 | F: CGATACCTACACGCCCCAGT |
|  | R: CAGGCTTTGTCCGAAGAG |
| BtAsiaII1-CSP9 | F: CGTGGTTGTTCTGGTCTG |
|  | R: TGATTCTTGCGGAGTTGG |
| BtAsiaII1-CSP10 | F: GTCGGCAGTTTCATCGTC |
|  | R: TTCCTGTGGGATCGTATTC |
| BtAsiaII1-CSP11 | F: GTTATCCTCGGGCAGTTAAG |
|  | R: AAAGTCATCCACGTCTCTCG |
| BtAsiaII1-CSP12 | F: GTTGGTTACTTCGCCTGGTG |
|  | R: ATTCCTTCGGCTTCTTCG |
| BtAsiaII1-CSP13 | F: GAGCAAGTACGACAACTTCG |
|  | R: TAGTGACCTTGGTGGTTTCC |
| BtAsiaII1-CSP14 | F: TTGTCTTGGTCGGCTGTG |
|  | R: TCGGGGTCGTATTTCTGC |

**Table. S4: Primers used for qPCR analysis for selected BtAsiaII1 OBP**

| BtAsiaII1-OBP1F | CTAGCTCAGATTCAGGGAAGTG |
| --- | --- |
| BtAsiaII1-OBP1R | GACCAAGCCTTGTACAGAGAA |
| BtAsiaII1-OBP2F | GGAATCTACAGGGAAGCCAAA |
| BtAsiaII1-OBP2R | CGGCTCCAGATTTACATTCCT |
| BtAsiaII1-OBP3F | CACAGAGGAGTGGCAACAAA |
| BtAsiaII1-OBP3R | CCGAGATAGGGTTGCACTTAAC |
| BtAsiaII1-OBP4F | GCCACGATGTCTTGAAGATTG |
| BtAsiaII1-OBP4R | GGCTCGGCAAACTTGTTATC |
| BtAsiaII1-OBP5F | TCCGCAACTACATGAACTGC |
| BtAsiaII1-OBP5R | TCCATTCCTCCGGGTATTCC |
| BtAsiaII1-OBP6F | GACGGTCAACAACACGAACA |
| BtAsiaII1-OBP6R | GTTCTCTGGGTACTGTGCCT |
| BtAsiaII1-OBP7F | TTGTGCCTGTCGGTCTTACT |
| BtAsiaII1-OBP7R | CTCAGTTGGGGAGCATTGTG |
| BtAsiaII1-OBP8F | GACAAAGCAAAGCAGGTCATAG |
| BtAsiaII1-OBP8R | CTTCAGTTACCACGCACTCT |
| BtAsiaII1-OBP9F | GGCCGAATTTTACACCTCCC |
| BtAsiaII1-OBP9R | ATGTCATCACTCTTCGGGCA |
| BtAsiaII1-OBP10F | GCCTGCCCTCTAGTTGAAAT |
| BtAsiaII1-OBP10R | GTGTATGGAGCCGTCACTTT |
| BtAsiaII1-OBP11F | GGGCTAAGAGGCTGATGATG |
| BtAsiaII1-OBP11R | CGTGGTTGGATTCACTTCTTTG |
| BtAsiaII1-OBP12F | CAAAGACGATGAAGGAGGCG |
| BtAsiaII1-OBP12R | ACCAACAGCGCAATGAACTT |
| BtAsiaII1-OBP13F | TCGCCAACTACATCAAGTGC |
| BtAsiaII1-OBP13R | TGTGGGTCGTACTTCTTGCT |
| BtAsiaII1-OBP14F | TAGTTGTCTTGGTCGGCTGT |
| BtAsiaII1-OBP14R | GGTTTTCAAAGCGTCGGGAA |

**Table. S5: Primers used for qPCR analysis for selected BtAsiaII1 CSP**

| BtAsiaII1-CSP1F | ACGAGGGACGTACCCTTAAA |
| --- | --- |
| BtAsiaII1-CSP1R | GGGTCTGGTAGAACTTGATGAC |
| BtAsiaII1-CSP2F | AGTGAACTCAAACGTCTCCTC |
| BtAsiaII1-CSP2R | CTTCCATTCGGTCGGGTATT |
| BtAsiaII1-CSP3F | CCTGATGCTCTATCCACCAAG |
| BtAsiaII1-CSP3R | CAATTTCGCCCACATTTCAG |
| BtAsiaII1-CSP4F | GCATTCTGTCCAACAAGAG |
| BtAsiaII1-CSP4R | ATCAGCACACTGCGTTTC |
| BtAsiaII1-CSP5F | TTGTGTTGTGCGTAGTGG |
| BtAsiaII1-CSP5R | TTCAGTTAGTTGCGGATGAG |
| BtAsiaII1-CSP6F | TCAAGGCGAAGAAGAGGA |
| BtAsiaII1-CSP6R | CGATCTTCTGGATGTTGGAC |
| BtAsiaII1-CSP7F | TCTTACTGGCGGAGGCTAT |
| BtAsiaII1-CSP7R | ATTTGAGCTGGCGCATGA |
| BtAsiaII1-CSP8F | CCGTAAGGTCATCAAGTTCTAC |
| BtAsiaII1-CSP8R | TACAGGGTTTGTCCGAAGA |
| BtAsiaII1-CSP9F | TTTCCGTGGTTGTTCTGG |
| BtAsiaII1-CSP9R | TTCAAGATGCTCTCGATGTC |
| BtAsiaII1-CSP10F | GACATCGACAGTGTTCTCAAG |
| BtAsiaII1-CSP10R | GCGCAAGTTGTTTCCAAAG |
| BtAsiaII1-CSP11F | GATGAAGGTCCTTGCACTAAC |
| BtAsiaII1-CSP11R | CATAACTGTGCGCACTATCC |
| BtAsiaII1-CSP12F | CCTCAACAACGATCGGATAC |
| BtAsiaII1-CSP12R | CACCTTGATTCCACCAACA |
| BtAsiaII1-CSP13F | AGGAGTGGACGAGAAACT |
| BtAsiaII1-CSP13R | CCATGAGGCACTTGATGTAG |
| BtAsiaII1-CSP14F | CCATCCTCAAGAACGAGAAG |
| BtAsiaII1-CSP14R | TTTCAAAGCGTCGGGAAG |

**Table. S6: showing the identified domain and its function found in OBPs of *B. tabaci* Asia II-1**

| **Gene** | **hit type** | **From** | **To** | **E-value** | **Short name** | **GO function** |
| --- | --- | --- | --- | --- | --- | --- |
| BtAsiaII1-OBP1 | Super family | 19  31 | 132  133 | 2.3e-10  15.4 | PBP_GOBP superfamily  PhBP domain | odorant binding([GO:0005549](http://www.ebi.ac.uk/QuickGO/GTerm?id=GO:0005549))  Pheromone binding  (GO:0005550) |
| BtAsiaII1-OBP2 | Super family | 101  217  106 | 219  249  210 | 0.0000075  1100  20.2 | PBP_GOBP superfamily  PhBP domain | odorant binding([GO:0005549](http://www.ebi.ac.uk/QuickGO/GTerm?id=GO:0005549))  Pheromone binding  (GO:0005550) |
| BtAsiaII1-OBP3 | Super family | 60  162 | 198  245 | 0.069  31 | PBP_GOBP superfamily | odorant binding([GO:0005549](http://www.ebi.ac.uk/QuickGO/GTerm?id=GO:0005549)) |
| BtAsiaII1-OBP4 | Super family | 17  29 | 132  133 | 2e-13  0.0585 | PBP_GOBP superfamily  PhBP domain | odorant binding([GO:0005549](http://www.ebi.ac.uk/QuickGO/GTerm?id=GO:0005549))  Pheromone binding  (GO:0005550) |
| BtAsiaII1-OBP5 | Super family | 38  27  86 | 201  68  202 | 7e-8  620  1.13 | PBP_GOBP superfamily  PhBP domain | odorant binding([GO:0005549](http://www.ebi.ac.uk/QuickGO/GTerm?id=GO:0005549))  Pheromone binding  (GO:0005550) |
| BtAsiaII1-OBP6 | Super family | 25  39 | 137  138 | 1.1e-14  0.0001 | PBP_GOBP superfamily  PhBP domain | odorant binding([GO:0005549](http://www.ebi.ac.uk/QuickGO/GTerm?id=GO:0005549))  Pheromone binding  (GO:0005550) |
| BtAsiaII1-OBP7 | Super family | 128  131 | 229  230 | 2.6e-8  7.39 | PBP_GOBP superfamily  PhBP domain | odorant binding([GO:0005549](http://www.ebi.ac.uk/QuickGO/GTerm?id=GO:0005549))  Pheromone binding  (GO:0005550) |
| BtAsiaII1-OBP8 | Super family | 31  45 | 147  148 | 1.1e-13  2.68e-05 | PBP_GOBP superfamily  PhBP domain | odorant binding([GO:0005549](http://www.ebi.ac.uk/QuickGO/GTerm?id=GO:0005549))  Pheromone binding  (GO:0005550) |
| BtAsiaII1-OBP9 | Superfamily | 18  135  25 | 138  167  139 | 4.00e-06  740  17.8 | PBP_GOBP superfamily  PhBP domain | odorant binding([GO:0005549](http://www.ebi.ac.uk/QuickGO/GTerm?id=GO:0005549))  Pheromone binding  (GO:0005550) |
| BtAsiaII1-OBP10 | Super family | 34  153 | 126  211 | 5.3e-30  4400 | OS-D superfamily/OBP A10 | Probably: membrane (GO:0016020) |
| BtAsiaII1-OBP11 | Superfamily | 1  7 | 105  102 | 5e-11  1.3 | PBP_GOBP superfamily  PhBP domain | odorant binding([GO:0005549](http://www.ebi.ac.uk/QuickGO/GTerm?id=GO:0005549))  Pheromone binding  (GO:0005550) |
| BtAsiaII1-OBP12 | Super family | 21  33 | 134  135 | 2.4e-10  15.4 | PBP_GOBP superfamily  PhBP domain | odorant binding([GO:0005549](http://www.ebi.ac.uk/QuickGO/GTerm?id=GO:0005549))  Pheromone binding  (GO:0005550) |
| BtAsiaII1-OBP13 | Super family | 34  153 | 126  211 | 5.3e-30  4400 | OS-D superfamily | Probably: membrane (GO:0016020) |
| BtAsiaII1-OBP14 | Super family | 38  29  86 | 201  68  202 | 6.9e-8  610  1.13 | PBP_GOBP superfamily  PhBP domain | odorant binding([GO:0005549](http://www.ebi.ac.uk/QuickGO/GTerm?id=GO:0005549))  Pheromone binding  (GO:0005550) |

**Table. S7: showing the identified domain and its function found in CSPs of *B. tabaci* Asia II-1**

| **Gene** | **hit type** | **From** | **To** | **E-value** | **Short name** | **GO function** |
| --- | --- | --- | --- | --- | --- | --- |
| BtAsiaII1-CPS1 | Super family | 23 | 115 | 9.4e-37 | OS-D superfamily | Probably: membrane (GO:0016020) |
| BtAsiaII1-CSP2 | Super family | 25 | 117 | 4.7e-35 | OS-D superfamily | Probably: membrane (GO:0016020) |
| BtAsiaII1-CSP3 | Super family | 33 | 125 | 2.9e-37 | OS-D superfamily | Probably: membrane (GO:0016020) |
| BtAsiaII1-CSP4 | Super family | 29 | 120 | 1e-28 | OS-D superfamily | Probably: membrane (GO:0016020) |
| BtAsiaII1-CSP5 | Super family | 25 | 117 | 4e-33 | OS-D superfamily | Probably: membrane (GO:0016020) |
| BtAsiaII1-CSP6 | Super family | 34 | 126 | 5.3e-30 | OS-D superfamily | Probably: membrane (GO:0016020) |
| BtAsiaII1-CSP7 | Super family | 31 | 112 | 6.6e-14 | OS-D superfamily | Probably: membrane (GO:0016020) |
| BtAsiaII1-CSP8 | Super family | 23 | 115 | 2.7e-37 | OS-D superfamily | Probably: membrane (GO:0016020) |
| BtAsiaII1-CSP9 | Superfamily | 25 | 117 | 2.8e-33 | OS-D superfamily | Probably: membrane (GO:0016020) |
| BtAsiaII1-CSP10 | Super family | 25 | 117 | 6.4e-34 | OS-D superfamily | Probably: membrane (GO:0016020) |
| BtAsiaII1-CSP11 | Superfamily | 33 | 124 | 5.9e-26 | OS-D superfamily | Probably: membrane (GO:0016020) |
| BtAsiaII1-CSP12 | Super family | 40 | 141 | 6.1e-26 | OS-D superfamily | Probably: membrane (GO:0016020) |
| BtAsiaII1-CSP13 | Super family | 27 | 118 | 1.7e-35 | OS-D superfamily | Probably: membrane (GO:0016020) |
| BtAsiaII1-CSP14 | Super family | 25 | 117 | 4.7e-35 | OS-D superfamily | Probably: membrane (GO:0016020) |

**Table. S8: Localization and clustering of OBP genes across the chromosomes of the *Bemisia tabaci***

| **Chromosome number** | **OBP genes** | **Name of OBP gene** | **Percentage of genes in clusters** |
| --- | --- | --- | --- |
| 3 | 4 genes in cluster 1  2 gens in cluster 2 | OBPs 1, 6, 8 & 12  OBP 5 &14 | 28.57 % of genes in cluster1  14.28 % of OBP genes in cluster 2 |
| 2 | 2 genes in cluster 3 | OBP 2 and 9 | 14.28 |
| 6 | 2 genes in cluster 4 | OBP 7, 10 and 13 | 14.28 |
| 10 | 2 genes in cluster 5 | OBP 4 and 11 | 14.28 |

*B. tabaci* Q genome Bioproject ID: PRJEB47898 was used as a model for annotation

**Table. S9: Localization and clustering of CSP genes across the chromosomes of the *Bemisia tabaci***

| **Chromosome number** | **CSP genes** | **Name of CSP gene** | **Percentage of genes in clusters** |
| --- | --- | --- | --- |
| 6 | 5 genes in cluster 1 | CSPs 2,3,6,11 and 14 | 35.71 of genes in cluster 1 |
| 7 | 4 genes in cluster 2 | CSPs 1, 8, 9 and 10 | 28.57 of genes in cluster 2 |
| 9 | No cluster | CSP 4 | 0% |
| 4 | No cluster | CSP 7 | 0% |
| 8 | No cluster | CSP 13 | 0% |

*B. tabaci* Q genome Bioproject ID: PRJEB47898 was used as a model for annotation

**Table. S10: Details of ligands used for molecular docking in this study**

| Sl. No. | **Compound name** | **Chemical structure** | **Molecular formula** | **Role** | **Target insect species** | **References** |
| --- | --- | --- | --- | --- | --- | --- |
| 1 | beta-Ionone | 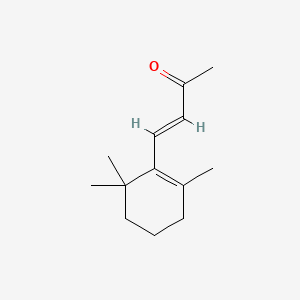 | C_13_H_20_O | Oviposition site/ host selection | *B. tabaci* | Li et al., 2019 and Wang et al., 2019 |
| 2 | p-cymene | 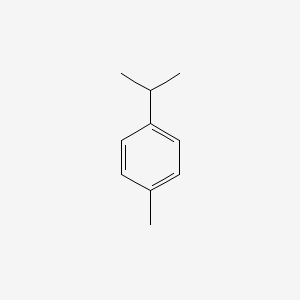 | C_10_H_14_ | Oviposition deterrents | *Plutella xylostella* | Wang et al., 2008 |
| 3 | beta-Ocimene | 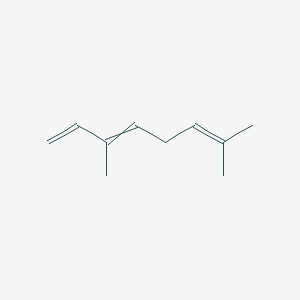 | C_10_H_16_ | Attractant | *Aphis gossypii and Hyphantria cunea* | Mauck et al., 2010; Tang et al., 2012 |
| 4 | beta-caryophyllene | 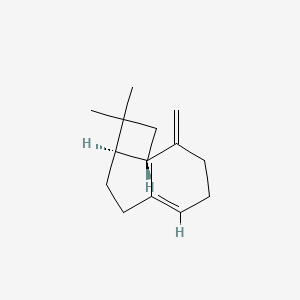 | C_15_H_24_ | Attractant | *Trialeurodes vaporariorum* | Darshanee et al., 2017 |
| 5 | NeoPhytadiene | 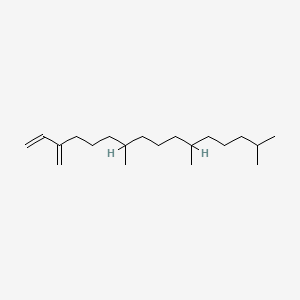 | C_20_H_38_ | Attractant | *B. tabaci (MED)* | Shi et al., (2019) |
| 6 | Myrcene | 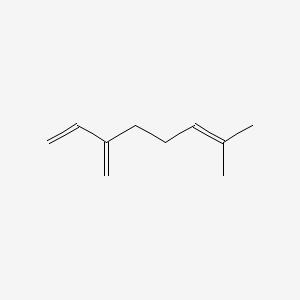 | C_10_H_16_ | attractant | *Encarsia Formosa* | Liu et al., 2017 |
| 7 | beta-Pinene | 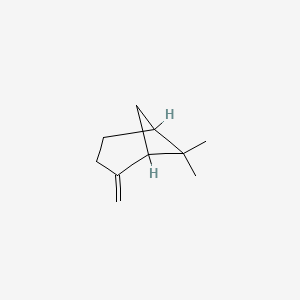 | C_10_H_16_ | Repellent | *Tribolium castaneum* | Pajaro-Castro et al., 2017 |
| 8 | alpha-pinene | 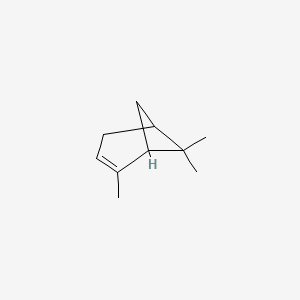 | C_10_H_16_ | Repellent | *B. tabaci* | Chen et al., 2017 |
| 9 | cis-3-Hexen-1-ol | 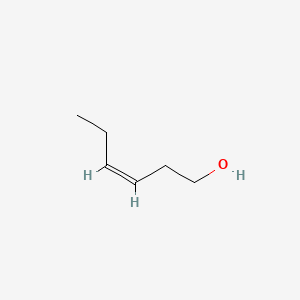 | C_6_H_12_O | attractant | *Trialeurodes vaporariorum* | Darshanee et al., 2017 |
| 10 | Limonene | 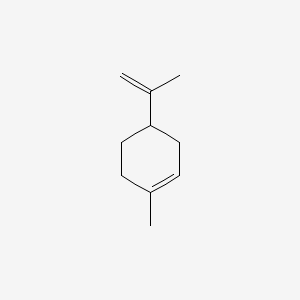 | C_10_H_16_ | Repellent | *Tribolium confusum* | Malacrinò et al., 2016 |
| 11 | Azulene | 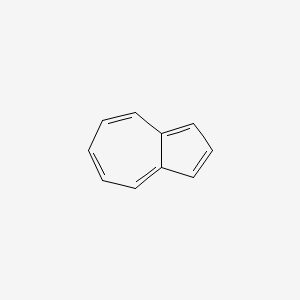 | C_10_H_8_ | Repellent/Attractant | *Aedes aegypti/Ceratitis capitata* | Stappen et al., 2021 |

**Fig S1. Structure validation report of protein A) OBP8 and B) CSP4.** The structural validation was done using the criteria to identify the Ramachandran outliers (detecting unfavourable backbone conformations), recognising sidechain outliers (flagging problematic sidechain conformations), highlighting RSRZ outliers (indicating atypical stereochemical properties), quantifying steric clashes through clashscore assessment, and evaluating the quality of model fit to experimental data via Rfree measurements.


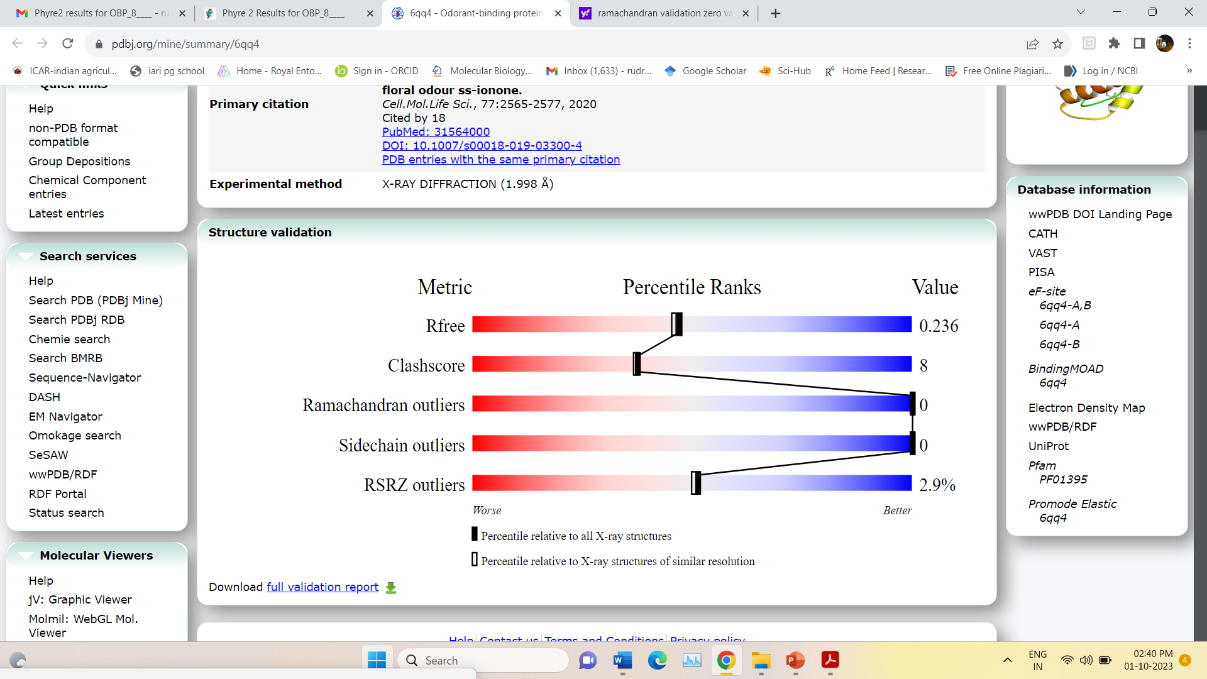

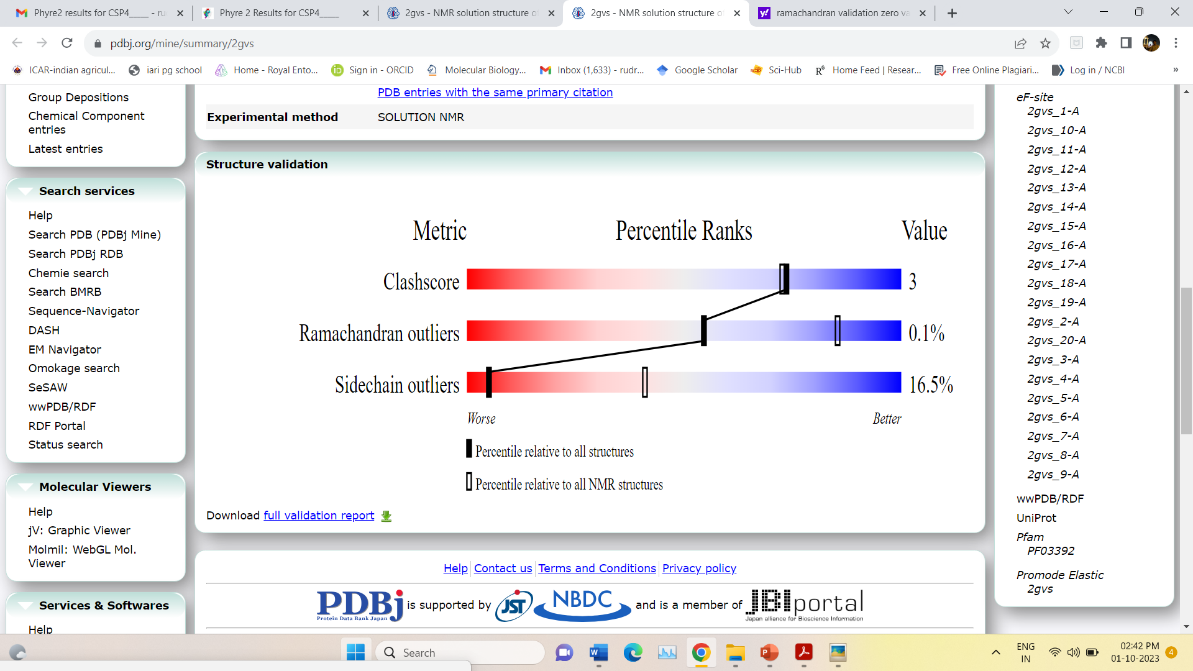


**A B**

**Fig S2. Motifs identified in OBPs of Bemisia tabaci AsiaII-1.** Motifs were discovered by using the MEME tool. The heights of the symbols within a stack indicate the relative frequency of the amino acids at that position. Parameters used for motif discovery were: minimum width = 6, maximum width = 10, maximum number of motifs to find = 6. The numerical value in the bracket indicates the E-value for each motif

**
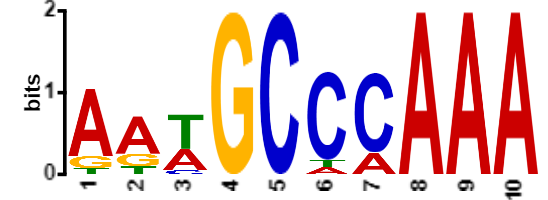

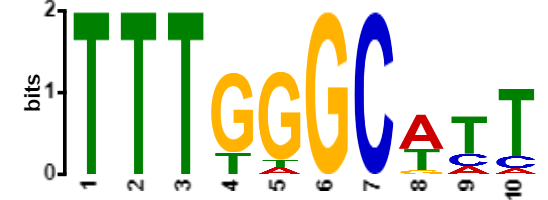
**

**
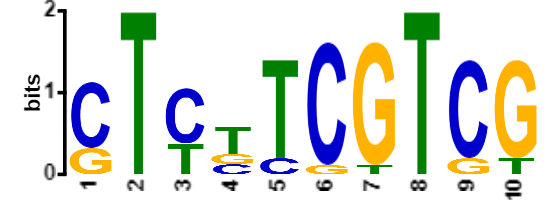

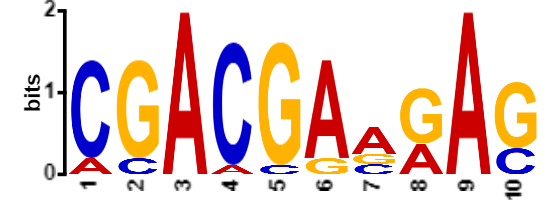
**

**
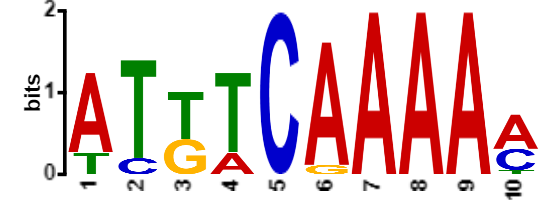

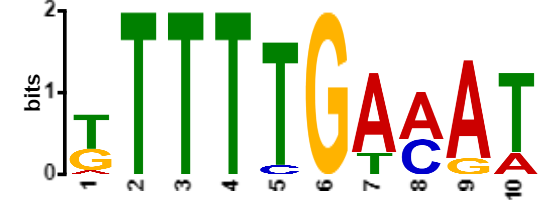
**

**
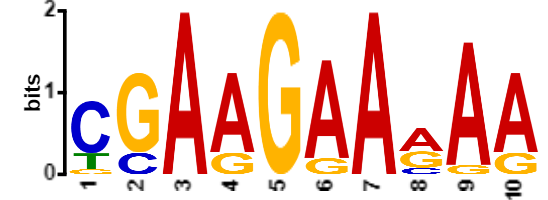

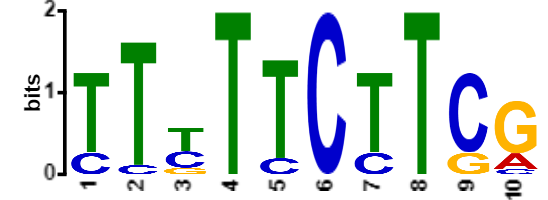
**

**
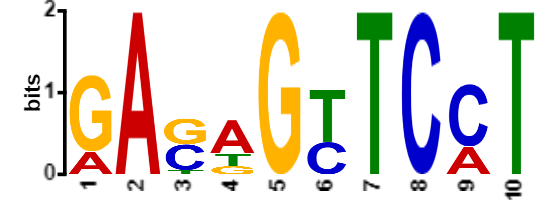

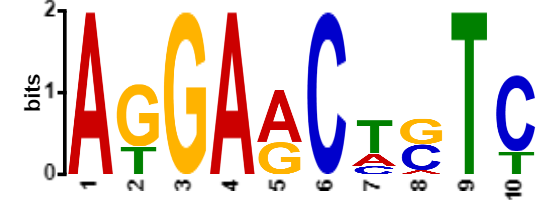
**

**
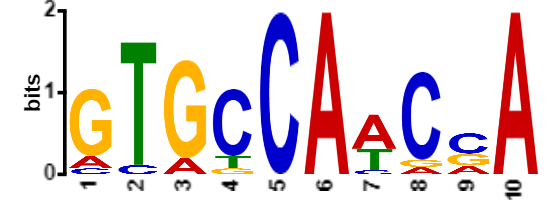

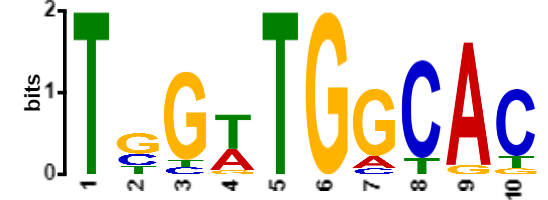
**

**Fig S3. Motifs identified in CSPs of *Bemisia tabaci* Asia II-1.** Motifs were discovered by using the MEME tool. The heights of the symbols within a stack indicate the relative frequency of the amino acids at that position. Parameters used for motif discovery were: minimum width = 6, maximum width = 10, maximum number of motifs to find = 6. The numerical value in the bracket indicates the E-value for each motif

**
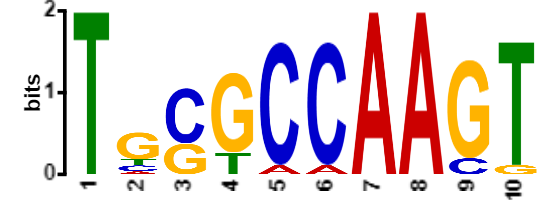

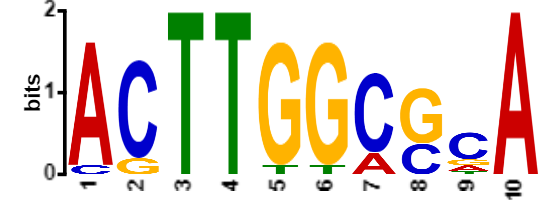
**

**
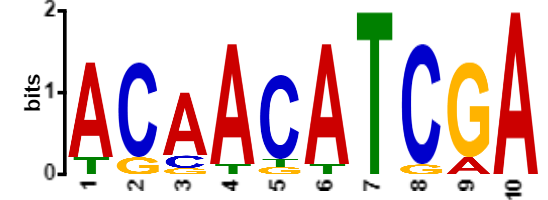

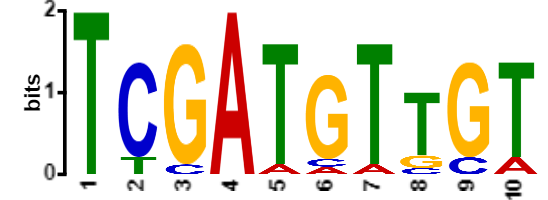
**

**
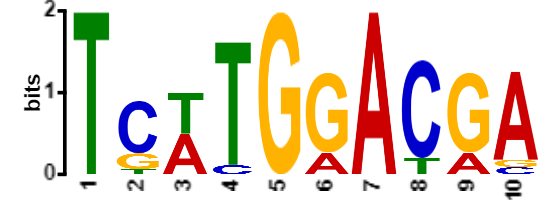

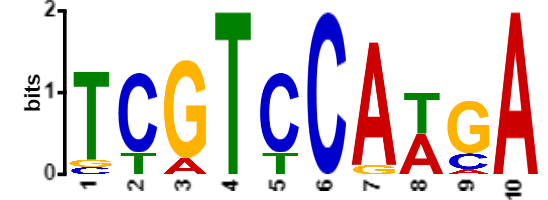
**

**
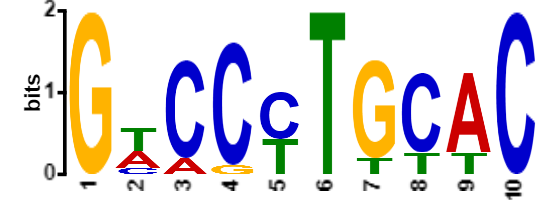

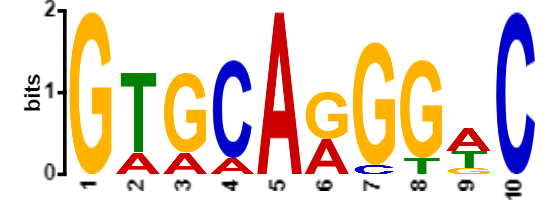
**

**
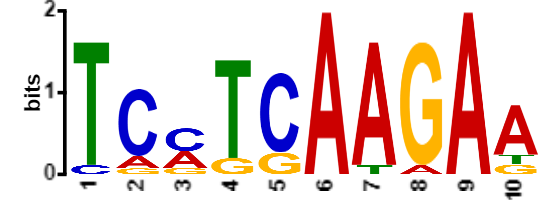

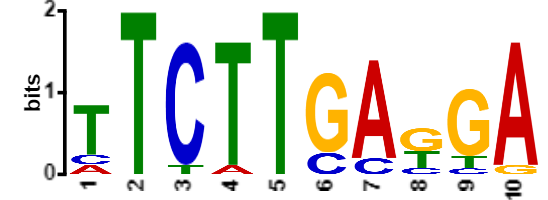
**

**
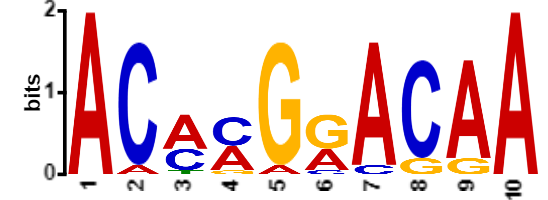

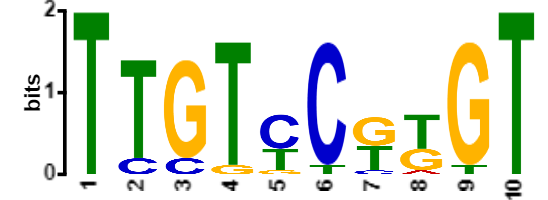
**

**Fig S4. Transcript levels of the *B. tabaci* Asia II-1 OBP8 and CSP4 genes. Expression levels of these two genes were evaluated by qRT-PCR at different developmental stages. The standard error is represented by the error bar.**

**Fig S5. Phylogenetic study of *Bemisia tabaci* Asia II-1's amino acid sequences in relation to other hemipteran odorant-binding proteins (OBPs).** neighbor-joining tree was created for the odorant-binding proteins (OBPs). The 1000 replications used to obtain the bootstrap values are indicated on the nodes. The 142 OBPs used in this investigation are included in a supplemental file along with their protein names and sequences. Apis = *Acyrthosiphon pisum*,, Agos = *Aphis* *gossypii*, Afab = *Aphis fabae*, Agly = *Aphis glycines*, Aluc = *Apolygus lucorum*, Alin = *Adelphocoris lineolatus*, Acra = *Aphis craccivora*, BtAsiaII1= *Bemisia* *tabaci* Asia II-1, BtabMED = *Bemisia tabaci*MED, BtabMEAM1= *Bemisia* *tabaci*MEAM1, Bbra = *Brevicoryne brassicae*, Dpla = *Drepanosiphum platanoidis*, Lery = *Lipaphis erysimi*, Mper = *Myzus persicae*, Mdir = *Metopolophium dirhodum*, Mvic = *Megoura viciae*, Nlug = *Nilaparvata lugens*, Nrib = *Nasonovia ribis-nigri*. Psal = *Pterocomma salicis*, Rpad = *Rhopalosiphum padi*, Sfur = *Sogatella furcifera* and Tsal = *Tuberolachnus salignus*


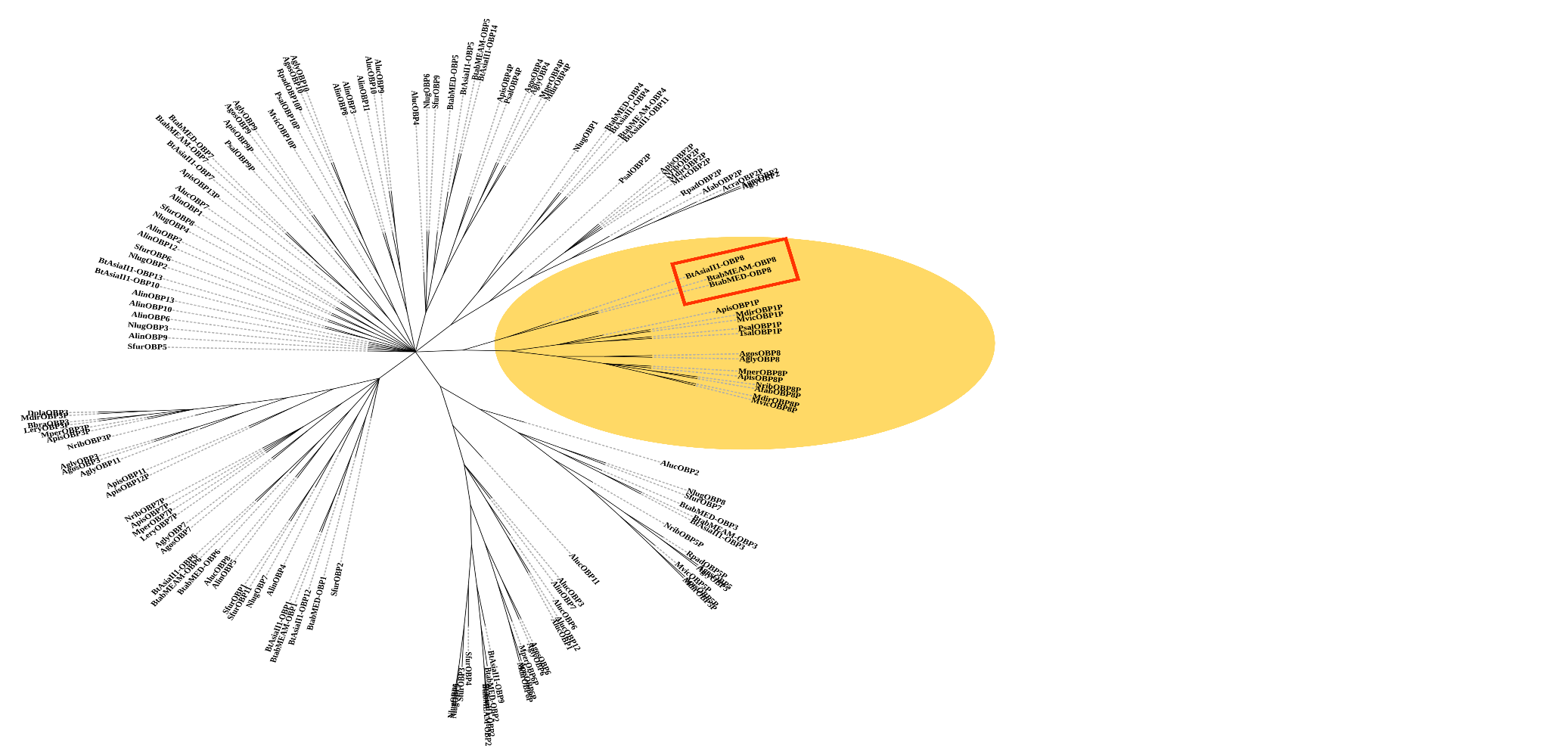


**Fig S6. Phylogenetic study of *Bemisia tabaci* Asia II-1's amino acid sequences in relation to other hemipteran chemosensory proteins (CSPs).** The neighbor-joining tree was created for the chemosensory proteins (OBPs). The 1000 replications used to obtain the bootstrap values are indicated on the nodes. The 48 CSPs used in this investigation are included in a supplemental file along with their protein names and sequences. Adelphocoris lineolatus is represented by Alin, Aphis gossypii by Agos, Apolygus lucorum by Aluc, Myzus persicae by Mper, Nilaparvata lugens by Nlug, Sogatella furcifera by Sfur, and Bemsia tabaci by Btab


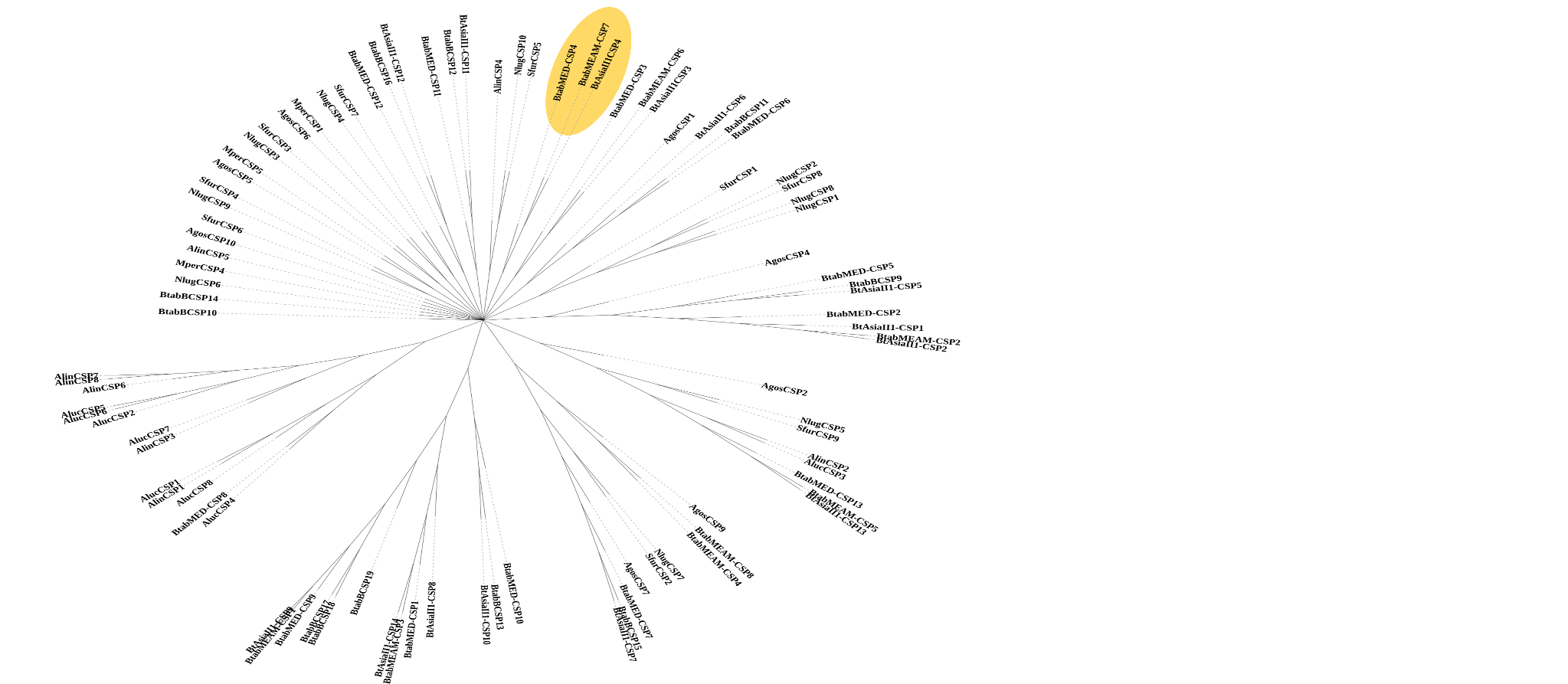


**Fig S7. Phylogeneic tree of OBPs across *B. tabaci* cryptic species**


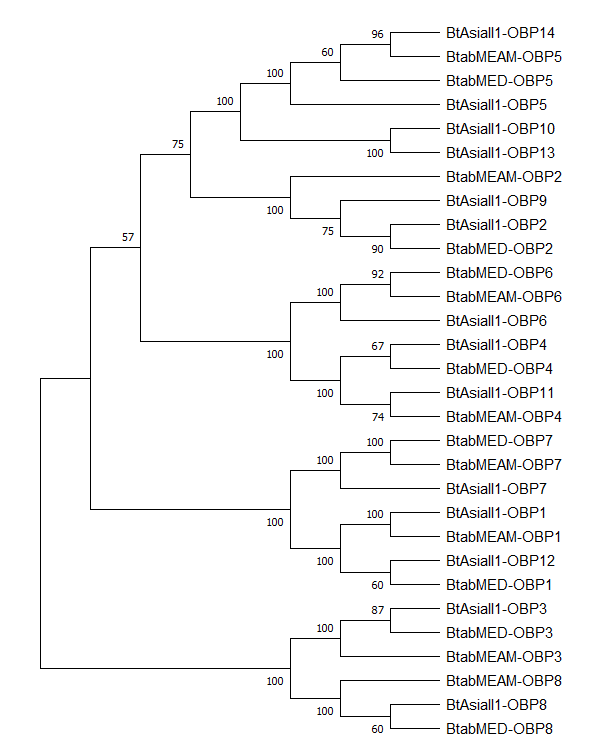


**Fig S8. Phylogenic tree of CSPs across *B. tabaci* cryptic species**


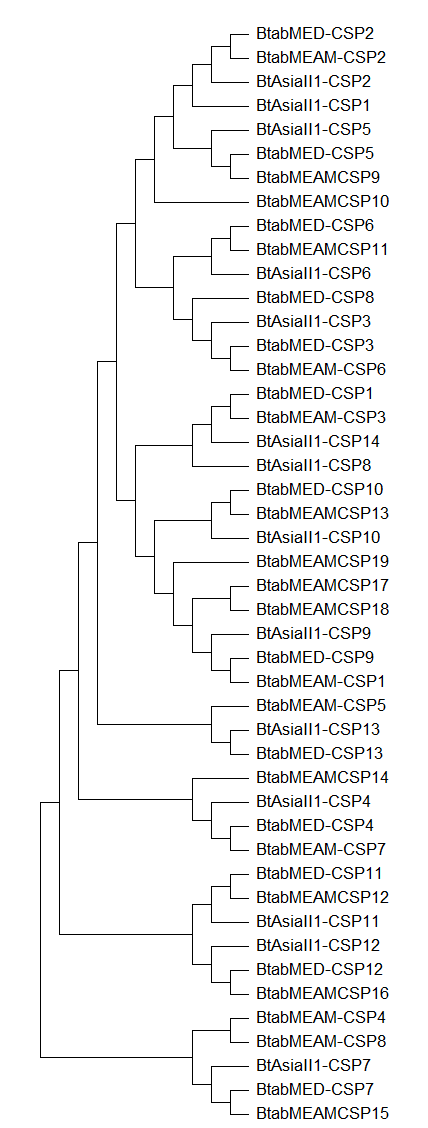

Supplement: Supplementary file 1 — Supplementary Information 1. [file 41598_2024_65785_MOESM1_ESM.docx]
